# Mosaic vaccination: How distributing different vaccines across a population could improve epidemic control

**DOI:** 10.1002/evl3.252

**Published:** 2021-08-23

**Authors:** David V. McLeod, Lindi M. Wahl, Nicole Mideo

**Affiliations:** ^1^ Centre D'Ecologie Fonctionnelle & Evolutive CNRS Montpellier 34090 France; ^2^ Mathematics Western University London ON N6A 5B7 Canada; ^3^ Department of Ecology and Evolutionary Biology University of Toronto Toronto ON M5S 3B2 Canada

**Keywords:** antigenic evolution, evolutionary epidemiology, mosaic therapy, vaccination

## Abstract

Although vaccination has been remarkably effective against some pathogens, for others, rapid antigenic evolution results in vaccination conferring only weak and/or short‐lived protection. Consequently, considerable effort has been invested in developing more evolutionarily robust vaccines, either by targeting highly conserved components of the pathogen (universal vaccines) or by including multiple immunological targets within a single vaccine (multi‐epitope vaccines). An unexplored third possibility is to vaccinate individuals with one of a number of qualitatively different vaccines, creating a “mosaic” of individual immunity in the population. Here we explore whether a mosaic vaccination strategy can deliver superior epidemiological outcomes to “conventional” vaccination, in which all individuals receive the same vaccine. We suppose vaccine doses can be distributed between distinct vaccine “targets” (e.g., different surface proteins against which an immune response can be generated) and/or immunologically distinct variants at these targets (e.g., strains); the pathogen can undergo antigenic evolution at both targets. Using simple mathematical models, here we provide a proof‐of‐concept that mosaic vaccination often outperforms conventional vaccination, leading to fewer infected individuals, improved vaccine efficacy, and lower individual risks over the course of the epidemic.

## Impact Summary

Evolutionary biologists and ecologists have long noted that variation in a host population can be protective against disease. Confronted with a specific pathogen threat, homogeneous populations may face one of two extreme scenarios. In the best case, all individuals are resistant to the pathogen, whereas in the worst case, all individuals are highly susceptible. The role of heterogeneity, therefore, is to hedge against the worst case at the cost of potentially failing to achieve the best case. Although this protective effect of host heterogeneity has received empirical support in some systems, human disease control measures often attempt to constrain variation. Indeed, vaccination aims to replicate the best‐case scenario above: all individuals fully resistant to a particular pathogen threat should provide the best population‐level protection. Yet, vaccination against some diseases (e.g., influenza) fails to achieve this ideal and would still fail even if 100% of individuals were vaccinated. This is because the targeted pathogens are themselves variable and rapidly evolving, and the immune responses elicited by vaccination against a particular strain are not fully protective against other circulating strains. Given that vaccination often fails to replicate the best‐case scenario for a homogeneous population, we used mathematical models to answer the question, should vaccination instead seek to exploit the benefits of a heterogeneous population? Our analysis demonstrates that creating a “mosaic” of immunity at the population level by distributing a set of qualitatively different vaccines (focused either on different strains or different antigens) often outperforms conventional vaccination, reducing the total number of infections and increasing estimates of vaccine efficacy.

After hygiene, vaccines are arguably the greatest success story in public health to date. Vaccines are responsible for the eradication of smallpox in humans and rinderpest in livestock, and have driven substantial declines in the incidence of numerous childhood illnesses. Between 2010 and 2015, an estimated 10 million lives were saved by vaccines (World Health Organization 2017). Although other control measures, like drugs, are failing in the face of pathogen evolution, vaccines seem comparatively robust (Kennedy and Read 2017, 2018).

Yet vaccines are not immune to the challenges posed by pathogen evolution. As a result of either high mutation rates or existing standing variation, many pathogen populations harbor diversity in relevant immune‐signaling sites. If that diversity translates to relatively weak immune responses against strains other than the one to which a host was previously exposed, then vaccines—often produced from one or a few target strains—will fail to offer broad protection. It is precisely this combination of limited cross‐immunity (i.e, protection against different strains), resultant strong selection (Smith et al. 2004; Xue and Bloom 2020), and rapid evolution that necessitates yearly updating of the composition of, for example, seasonal influenza vaccines.

Efforts are ongoing to improve vaccination strategies against evolving threats like influenza (Petrova and Russell 2018). In particular, the search for more highly conserved targets of immune responses may yet produce a universal vaccine that need not be updated in the face of antigenic evolution (Okuno et al. 1993; Fiers et al. 2009; Mallajosyula et al. 2014; Nachbagauer and Krammer 2017). Yet the consequences of such vaccines, including for pathogen evolution, have not been fully elucidated (Viboud et al. 2020). An alternative strategy is a vaccine cocktail, designed to elicit immune responses against multiple targets (i.e., epitopes) with a single vaccine (Viboud et al. 2020); much like drug cocktails, these are expected to be more robust in the face of evolution (REX Consortium 2013). Multi‐strain vaccines offer another kind of cocktail (e.g., eliciting immune responses against different variants of the same target), which has been broadly useful for limiting disease caused by some pathogens (e.g., pneumococcal vaccines; Hausdorff and Hanage 2016).

Here we investigate a different vaccination strategy by asking if and when vaccinating individuals with one of a number of qualitatively different vaccines—essentially a “cocktail” at the population level— produces better epidemiological outcomes than a strategy that gives every individual the same vaccine. More accurately, in the vernacular of resistance evolution, this would be a “mosaic” strategy (REX Consortium 2013). The epidemiological consequences of using a mosaic vaccination strategy, to our knowledge, have not been explored. We analyze a mathematical model to specifically ask, first, if vaccines with different targets existed, what would be the optimal way to use them? Second, we consider the scenario of having a set of vaccines that target the same immunologic site, but different genetic (and antigenic) variants, for which limited cross‐immunity may exist. Finally, we ask which source of variation across vaccine doses—targets or variants—can produce the best outcomes when vaccine escape is either likely or rare. Overall, using a simple model we demonstrate that conventional vaccination programs, in which all individuals receive identical vaccines, are often outperformed by mosaic strategies that deliberately seed variation in the host population.

## The model

Here we provide an overview of the model; all mathematical details can be found in the Supporting Information. Consider a pathogen modeled in a standard SIR framework. Infection leads to sufficiently broad and long lasting immunity such that once infected, hosts cannot be reinfected over the timescale under consideration. Let R denote the basic reproductive ratio of the pathogen in an unvaccinated population.

**Figure 1:**
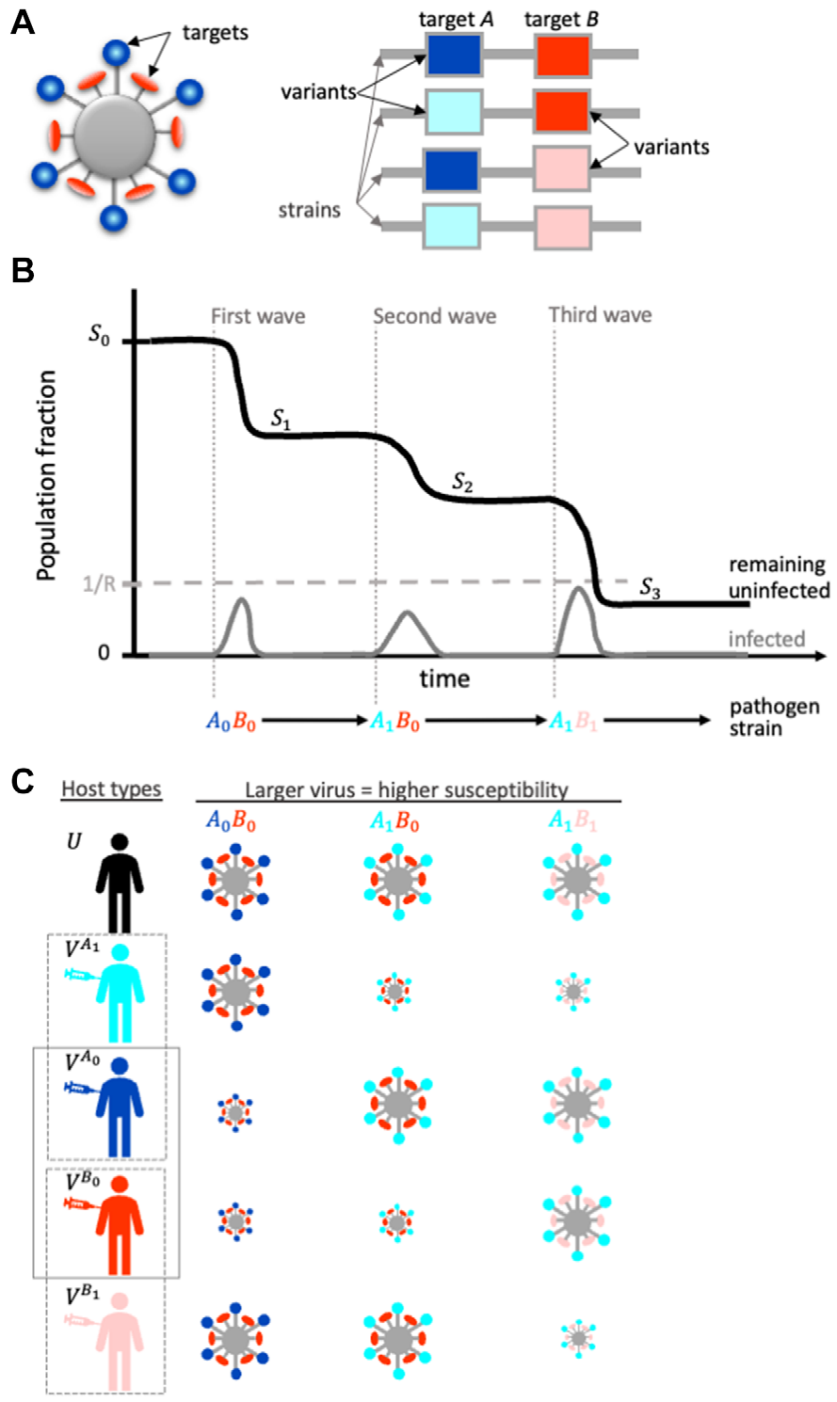
Model schematics. (A) Assumptions about pathogen structure (left) and genome (right); each line represents a unique pathogen genome, or “strain,” while the boxes represent specific loci. We use “targets” to describe different surface proteins or, equally, different epitopes on the same surface protein. We use “variants” to capture antigenically distinct versions of a given target (see text for details). In (B), we illustrate typical dynamics of our model, where $S_{n}$ denotes the fraction of susceptible individuals (black curve) remaining after the nth wave of the epidemic. The gray curve shows infected individuals, and captures our assumption that each wave reaches its epidemiological conclusion before another can begin, if the fraction of susceptibles is above 1/R. One possible epidemic sequence of strains is shown. (C) The impacts of different vaccination strategies. Hosts are either unvaccinated, U, or receive one of four possible candidate vaccines, which provide different levels of protection against the hypothetical strains in (B). Unlike conventional vaccination (e.g., $V^{A_{0}}$ only), distributing doses across targets ($V^{A_{0}}$ and $V^{B_{0}}$; solid box) provides strong protection against the initial strain and some protection against a subsequent strain; the consequences will depend on the precise allocation of doses between targets and the evolutionary trajectory of the pathogen; for the example shown in (B), greater allocation toward $V^{B_{0}}$ would be advantageous. In contrast, distributing across variants ($V^{A_{0}}$ and $V^{A_{1}}$ or $V^{B_{0}}$ and $V^{B_{1}}$; dashed boxes) reduces population protection against the initial strain in exchange for greater protection against a subsequent strain.

The pathogen has two potential vaccine “targets,” A and B, each of which may exhibit antigenic variation. Biologically, these targets could be different surface proteins, or different epitopes on the same surface protein. We use “variants” to refer to immunologically distinct versions of these targets (described in more detail below), and “strains” to refer to pathogens harboring different variants, such that $A_{i}B_{j}$ denotes the pathogen strain with variant i at target A and variant j at target B (see Fig. 1A). Antigenic space at each target is one dimensional (Lin et al. 2003), while antigenic change is cumulative and equally likely at either target. Thus if the initial pathogen strain is $A_{0}B_{0}$, then the next strain will be either $A_{1}B_{0}$ or $A_{0}B_{1}$ with equal probability. Antigenic change can be imported from an external source such as a reservoir animal population or a geographic region in which the pathogen is endemic (e.g., as for influenza A; Russell et al. 2008; Bedford et al. 2015), or a geographic region in which a new variant has recently been identified.

We treat pathogen evolution as a sequence of discrete antigenic changes, each of which may (or may not) produce a “wave” of the epidemic, such that the overall infection process consists of a series of strain‐specific waves (Fig. 1B). To simplify the mathematical analysis, we assume that these strain‐specific waves occur sequentially, rather than simultaneously, that is, a single strain dominates at any time. This assumption is valid provided antigenic change is sufficiently infrequent that by the time a novel strain has risen to appreciable levels in the population, the epidemic wave by the previous strain has largely concluded.

A fraction p of the population is vaccinated, and the vaccine doses can be distributed among four possible candidates (Fig. 1C). A fraction x of the p doses are distributed to vaccine target A, and 1−x to target B. Of the doses allocated to target A, a fraction $y_{A}$ are distributed to the most abundant variant in the population initially, $A_{0}$, and $1-y_{A}$ to $A_{1}$; likewise of the doses against B, a fraction $y_{B}$ will target $B_{0}$, while $1-y_{B}$ target $B_{1}$. Although we restrict vaccine distribution to the set $\left\{A_{0},A_{1},B_{0},B_{1}\right\}$, we make no such restriction for antigenic variation, and so it is possible that the variants $A_{k}$ and/or $B_{k}$, k>1 may emerge in the population. Note that we have assumed that the breadth of antigenic protection of any vaccine is limited, due to, for example, immune interference or safety considerations. Each target/variant combination in our model is thus defined by this limit; different variants are by definition antigens that escape the coverage of a single vaccine. Of course in reality, vaccines should be constructed to be as broadly cross‐protective as possible, provided there is no trade‐off with peak protection. Our basic model assumes that “universal” vaccines are not available but in Supporting Information S10.5 we investigate a trade‐off in which increasing the “broadness” of vaccine cross‐protection causes a concomitant reduction in “peak” protection.

Each vaccine reduces the probability of infection against its intended target/variant combination by a factor $\chi_{0}$, and more generally, reduces the probability of infection by a variant Δℓ antigenic units from its intended target/variant combination by a factor $\chi_{\Delta \ell }$. If vaccine cross‐protection were sufficiently broad, any antigenic variation would be irrelevant for vaccinated individuals, and so the allocation of vaccine doses would not matter. As cross‐protection decreases, however, antigenic variation will allow pathogens to escape vaccine protection and so how vaccine doses are distributed is increasingly important. In our model, the broadness of vaccine cross‐protection will depend on both the units of antigenic space and the timescale of interest, and so we focus on the case in which vaccine cross‐protection is negligible. This is done for simplicity, but we stress that the primary effect of broadening cross‐protection is to reduce the impact of vaccine distribution. Note that our model assumes the protection from naturally acquired immunity is superior (or no worse) to vaccine‐acquired immunity. The validity of this assumption will be disease‐ and vaccine‐specific: for example, it seems to hold for influenza (Kim et al. 2016; Chen et al. 2018; Viboud et al. 2020), but not SARS‐CoV‐2 (e.g., Greaney et al. 2021). We later consider the implications of waning naturally acquired immunity.

Let $S_{0}$ denote the initial density of individuals without infection‐acquired immunity (susceptibles). $S_{1}$ is then the density of susceptibles after the first wave (caused by the initial strain $A_{0}B_{0}$) and in general $S_{n}$ denotes the susceptible density following the nth wave. These individuals can be further divided based on whether they have been vaccinated. Let $U_{n}$ denote the density of susceptible, unvaccinated individuals, and $V_{n}^{\tau }$ denote the density of susceptible individuals vaccinated against target/variant combination τ (where τ can be $A_{0}$, $A_{1}$, $B_{0}$, or $B_{1}$), following the nth wave. Therefore,

(1) $$\begin{array}{c} S_{n}=U_{n}+\sum_{\tau }V_{n}^{\tau }. \end{array}$$

Suppose the initial densities are known, that is, $U_{0}=S_{0}\left(1-p\right)$ and the initial vaccine distribution is given. Then for a particular sequence of antigenic changes, the state of the population after each epidemic wave could be determined by numerically integrating the full SIR model. By rescaling time, however, we can directly compute the outcome of each wave using the approach described in the Supporting Information S3. In brief, for any sequence of antigenic changes, the remaining vaccinated individuals, after the nth wave, can be computed based only on the unvaccinated individuals remaining after each of the previous waves. To compute the unvaccinated individuals after each wave, $U_{i}$, we first determine whether the reproductive number of the pathogen strain with n antigenic changes is greater than 1. If it is less than or equal to 1, then $U_{n}$ is simply given by $U_{n-1}$, otherwise $U_{n}$ can be computed using a standard final size equation. All code used in numerical calculations and creating figures was written in MATLAB (MATLAB 2019), and has been uploaded as a Supporting File.

### THE LIKELIHOOD OF VACCINE ESCAPE

Given our assumption about the strength of immunity acquired through infection, there can be at most a single epidemic wave in an unvaccinated population. In a vaccinated population, however, antigenic change may cause successive waves as strains escape vaccine coverage. Our analysis will therefore focus on two representative scenarios of vaccine escape; in both we are interested in the expected remaining uninfected, denoted $E[S_{\text{final}}]$. In the first scenario, we assume that vaccine escape is rare, that is, at most one antigenic change occurs over the timescale of interest. Following the initial potential wave by strain $A_{0}B_{0}$, typically no antigenic change occurs, but there is a small probability that a single antigenic change occurs at either target with equal probability. Thus $E[S_{\text{final}}]$ is the average of these possibilities (Supporting Information S4).

In the second scenario, vaccine escape is common, and thus the sequence of epidemic waves is not limited by antigenic change. Specifically, following the initial potential wave by strain $A_{0}B_{0}$, the pathogen undergoes successive one unit antigenic changes (and successive potential waves) until the density of remaining individuals without infection‐acquired immunity is less than or equal to 1/R; at this point, the population will have herd immunity and any novel strain will be unable to cause an epidemic wave. In this case, $E[S_{\text{final}}]$ is the average remaining uninfected individuals computed over all possible antigenic sequences terminating in $S_{n}\leq 1/R$ (Supporting Information S4).

We take maximizing $E[S_{\text{final}}]$ as the metric for evaluating the efficacy of particular vaccination strategies. An important point to note is that when vaccine escape is common, the best possible outcome is $E[S_{\text{final}}]=1/R$. Thus irrespective of vaccine coverage, the population will ultimately be protected by herd immunity acquired through infection. The purpose of vaccination is then to “ease” the population gradually to the point of herd immunity so that when it is reached, there are few active infections in the population and “overshoot” (i.e., reducing the susceptible fraction to below 1/R) can be avoided (Handel et al. 2007). (NB: Overshoot occurs when herd immunity is reached while many active infections exist, even though, on average, infections are not able to replace themselves at this point, further transmissions occur as the wave declines.) In contrast, when vaccine escape is rare, considerably better outcomes than 1/R can be achieved.

Although we focus on choosing vaccination strategies that maximize $E[S_{\text{final}}]$, we are also interested in the consequences of these strategies for other metrics of vaccine success (Supporting Information S6). The first metric is vaccine efficacy, defined as

(2) $$\begin{array}{c} \text{VE}=1-\frac{\text{prob.}\;\text{of}\;\text{infection}\;\text{for}\;\text{vaccinated}\;\text{individual}}{\text{prob.}\;\text{of}\;\text{infection}\;\text{for}\;\text{unvaccinated}\;\text{individual}}. \end{array}$$

Here the probability of infection is computed over the entire epidemic. Vaccine efficacy measures the reduction in infection due to vaccination. Second, given an individual is both vaccinated and infected, what is the probability that they were infected by the strain they were vaccinated against (the “matched” strain)? We refer to this as vaccine matching, calculated as

(3) $$\begin{array}{c} \text{VM}=\frac{\#\;\text{of}\;\text{vaccinated}\;\text{individuals}\;\text{infected}\;\text{by}\;‘\text{matched}’\;\text{strain}}{\#\;\text{of}\;\text{vaccinated}\;\text{individuals}\;\text{infected}}. \end{array}$$

The importance of vaccine matching is that although our model assumes that the vaccine match determines only the probability of infection (i.e., $\chi_{\Delta \ell }$), there may be additional impacts including on the severity of symptoms and likelihood of hospitalization. Thus, all else being equal, a higher VM is desirable from a public health perspective.

### EPIDEMIC CASES

**Figure 2:**
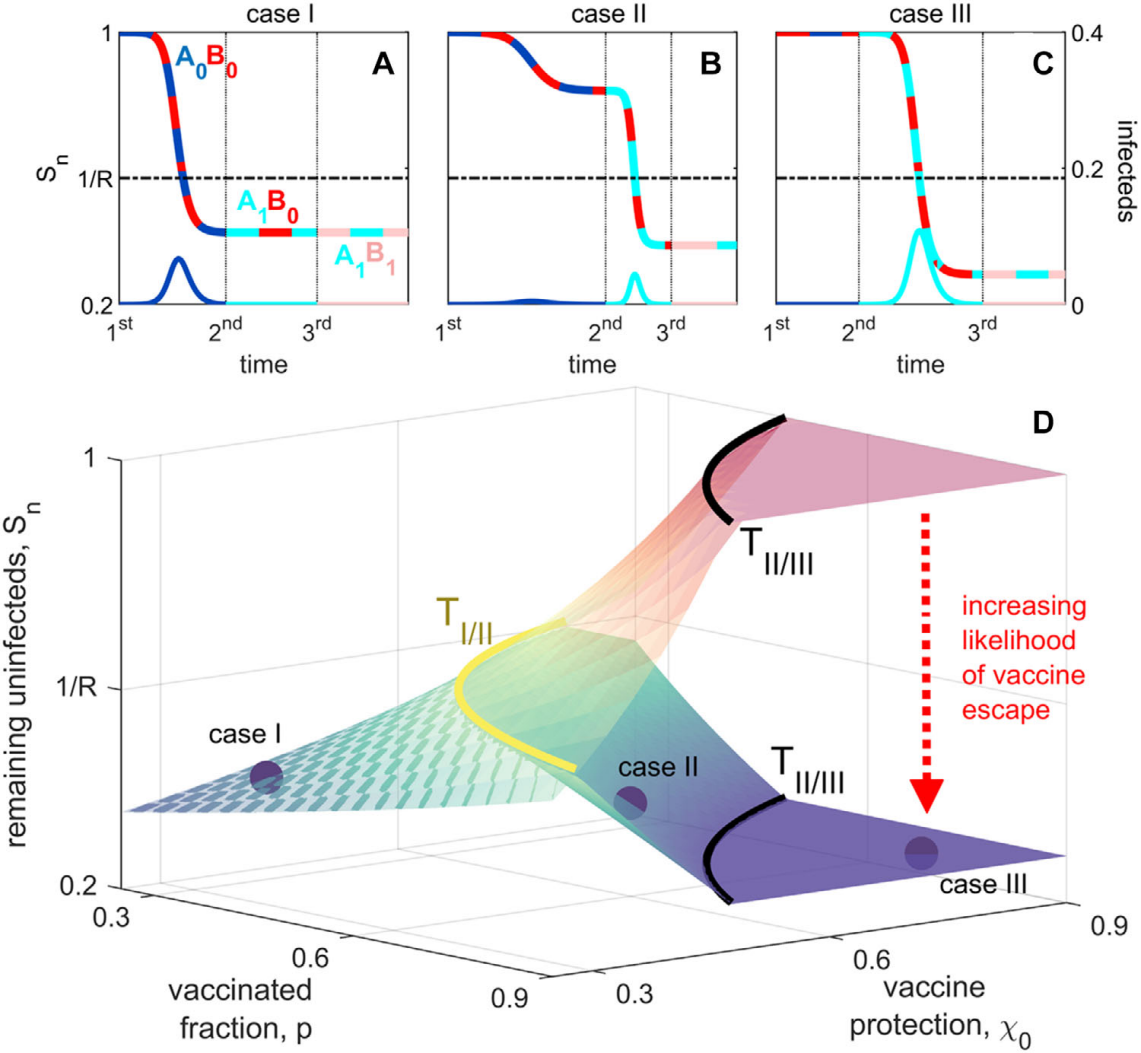
The three qualitatively different epidemic regimes under conventional vaccination. In case I, a single epidemic by the initial strain $A_{0}B_{0}$ occurs (A). In case II, there can be two epidemics, one by the initial strain, and one by the strain $A_{i}B_{j}$, here assumed to be $A_{1}B_{0}$ (B). In case III, the initial strain is blocked and a single epidemic wave occurs by a secondary (antigenically novel) strain (again, assumed here to be $A_{1}B_{0}$; C). In (D), we show the outcome in terms of the remaining uninfected individuals, $S_{n}$, as we vary vaccine coverage, p, and vaccine protection, $\chi_{0}$, where the upper translucent surface is the outcome achieved when vaccine escape does not occur, whereas the lower surface is the outcome achieved when vaccine escape is common. The curves delineating the thresholds between cases, $T_{\frac{I}{II}}\left(p\right)$ and $T_{\frac{II}{III}}\left(p\right)$, are shown for reference, while (A)–(C) show examples of the epidemic dynamics for each case. On the remaining susceptible curves, the two colors indicate the strain circulating in the population, whereas on the infecteds curves, the single color indicates what variant caused the epidemic wave. In all cases, R=1.75.

To provide a baseline for comparison, consider “conventional” vaccination, in which all doses are directed toward the primary variant of target A: x=1 and $y_{A}=1$. Here it is helpful to divide the space of epidemic outcomes into three qualitatively different cases, based on the expected outcome of conventional vaccination with vaccination coverage, p, and vaccine protection, $\chi_{0}$ (Fig. 2).

As illustrated in Figure 2A, case I occurs when protection is sufficiently weak that the initial wave reduces the density of susceptibles to below 1/R and no further waves can occur; we denote the vaccine protection threshold at which this occurs as $T_{\frac{I}{\text{II}}}\left(p\right)$. Thus when $\chi_{0}\leq T_{\frac{I}{\text{II}}}\left(p\right)$, infection‐acquired immunity (due to the large number of individuals who were infected) protects the population against further waves (Fig. 2A). Note that $T_{\frac{I}{\text{II}}}\left(p\right)$, and the scope of case I, has an intuitive dependence on R: as R increases, so too does the likelihood that a given $\chi_{0}$ and p will fall in case I. This is because increasing R increases pathogen transmissibility, so vaccines must be more protective (larger $\chi_{0}$) and vaccine coverage must be higher (larger p) in order for vaccines to have a substantial protective influence.

Case II occurs when coverage is of intermediate strength. As shown in Figure 2B, in case II the wave caused by the initial strain is such that $S_{1}>1/R$, and so antigenic change can generate subsequent waves until $S_{n}\leq 1/R$ (Fig. 2B). Thus, in case II, antigenic change is possible but not necessary for an epidemic. We denote the vaccine protection threshold for the upper limit of case II as $T_{\frac{\text{II}}{\text{III}}}\left(p\right)$, and thus case II occurs when $T_{\frac{I}{\text{II}}}\left(p\right)<\chi_{0}\leq T_{\frac{\text{II}}{\text{III}}}\left(p\right)$.

Finally, in case III, antigenic change is necessary for an epidemic to occur. As illustrated in Figure 2C, in case III vaccine protection is sufficiently strong that the reproductive number of the initial strain is less than 1 and so antigenic change at target A must occur before the epidemic proceeds, $\chi_{0}>T_{\frac{\text{II}}{\text{III}}}\left(p\right)$. Thus the first wave can only be caused by a strain with variant k≥1 at target A (Fig. 2C).

## Results

In what follows, we consider how to distribute vaccine doses against an evolving pathogen when vaccines have sufficient protection and coverage that antigenic escape has public health consequences (i.e., we are in case II or III). We summarize our key results in Table 1.

**Table 1:** Summary of results. $S_{\text{final}}$ denotes the remaining uninfected individuals ($E[S_{\text{final}}]$ in cases II and III); 1/R denotes the point at which the population attains herd immunity. Note that when the optimal strategy is a single allocation (e.g. case I between variants), the optimal strategy is conventional vaccination

	between targets (A_0_:B_0_)	between variants (A_0_:A_1_)
**Case I**: even with vaccination, A_0_B_0_ wave is severe and reduces S_final_ to below 1/R.	All allocations are equally effective.	Use A_0_ only.
**Case II**: vaccination is strong enough that A_0_B_0_ wave leaves S_final_ above 1/R. **Case III**: vaccination is strong enough that A_0_B_0_ wave is prevented.	**Cases II and III**: mosaic is always at least as good as (and often better than) conventional. See boxes below.	**Cases II and III**: mosaic can be better than conventional; outcome depends on escape probability. See boxes below.
Cases II and III, escape rare	A:B ratio has no effect on the first wave. Allocate to block one of the two possible second waves. Average outcome outperforms 50:50 allocation, and is always better than conventional vaccination.	If escape is very rare, maximize protection against A_0_: in Case II, allocate all doses to A_0_; in Case III, allocate sufficient doses to block the first wave, putting extra doses toward A_1_. As escape becomes more likely, balance shifts to maximizing protection against A_1_.
Cases II and III, escape common. Best outcome is S_final_ = 1/R.	Similar to above, allocate so that one of the possible wave sequences ends with S_final_ = 1/R. Although this makes things worse if the other mutation happens first, the average outcome outperforms 50:50 allocation, which outperforms conventional vaccination.	Unlike conventional vaccination, mosaic vaccination can always achieve S_final_ = 1/R in this case, after only one wave, or after two. To flatten the peak number of infections, allocate so that S_final_ = 1/R after the second wave.

### VACCINES DISTRIBUTED ACROSS TARGETS

**Figure 3:**
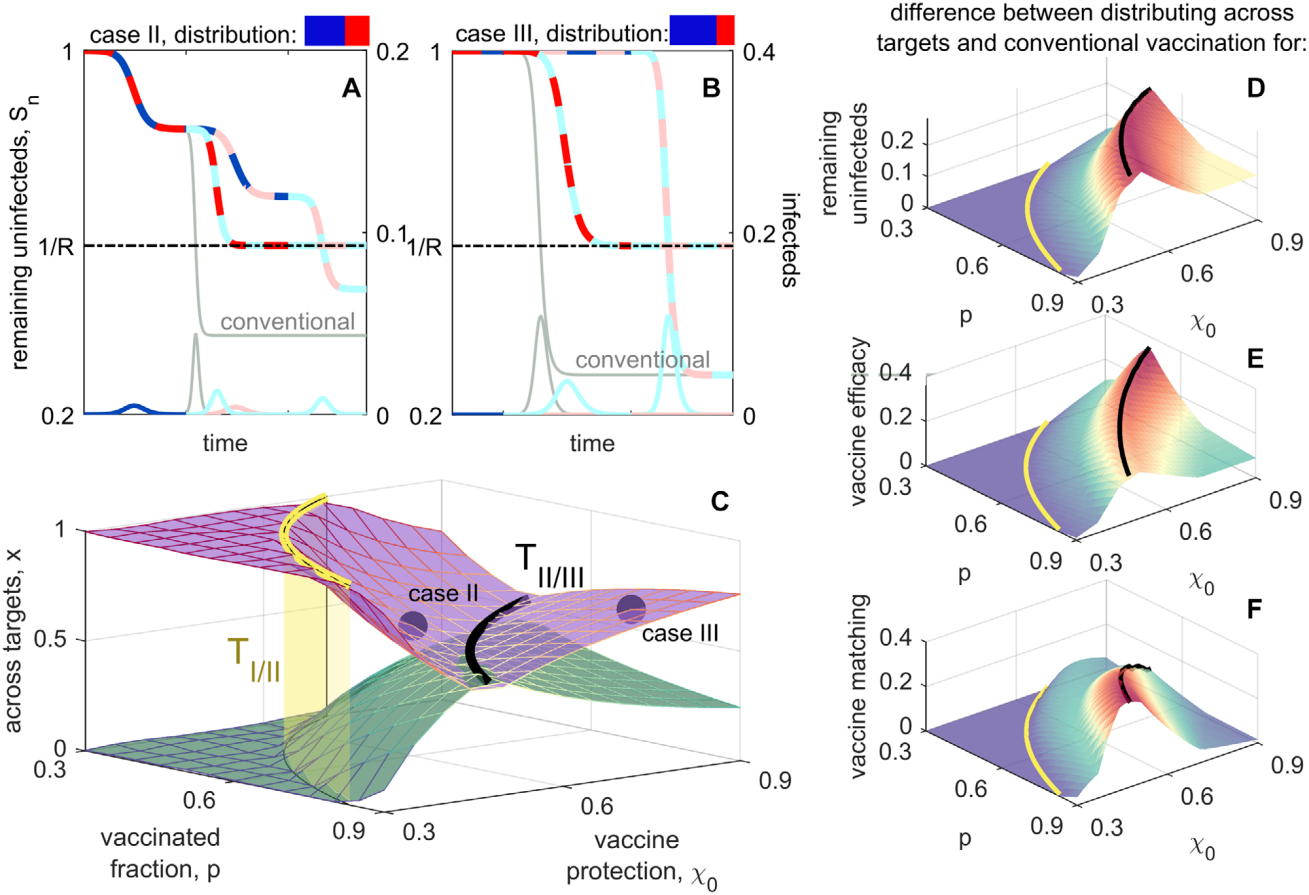
Optimal distribution of vaccines across targets when vaccine escape is common. (A and B) Example epidemic dynamics for case II (A) and case III (B), with conventional vaccination (light gray) shown for reference; colors used are as in Figure 2. Values of p, $\chi_{0}$, and the optimal distribution $x^{*}$ for these plots are indicated by the purple circles in (C). Following the initial epidemic wave by strain $A_{0}B_{0}$, there are two possible sequences of interest, $A_{0}B_{0}\to A_{1}B_{0}\to A_{1}B_{1}$ and $A_{0}B_{0}\to A_{0}B_{1}\to A_{1}B_{1}$; both are shown. On the remaining susceptible curves, the two colors indicate the strain circulating in the population, whereas on the infected curves, the single color indicates what variant caused the epidemic wave. (A) Mosaic vaccination is superior to conventional vaccination regardless of the sequence, whereas in (B) Mosaic vaccination is better on average and never worse. The optimal distribution across targets (shown in (C) as vaccine coverage, p, and protection, $\chi_{0}$, vary) maximizes the remaining susceptibles averaged over these sequences; when x is optimal, so is 1−x (purple, green). In (D)–(F), it is shown how distributing across targets outperforms conventional vaccination for three metrics; positive values indicate the degree to which mosaic vaccination is superior. (D) The difference in remaining uninfected, $S_{n}$. (E) The difference in vaccine efficacy, measured as attack rate over the course of the epidemic (Supporting Information 7). (F) The difference in vaccine matching, defined as the probability that an individual that is both vaccinated and infected is infected by a strain that they were vaccinated against. In all panels, the thresholds $T_{\frac{I}{II}}\left(p\right)$ (yellow surface) and $T_{\frac{II}{III}}\left(p\right)$ (black lines) are included for reference; beyond the yellow surface (i.e., further reducing p or $\chi_{0}$), vaccine efficacy is sufficiently low that evolution has no effect on the epidemic, and any distribution of vaccines between targets will produce the same outcome (for a given p and $\chi_{0}$). In all cases, R=1.75.

First, we consider distributing equally effective vaccines between two targets: a fraction x of available vaccine doses are used against target A and 1−x are used against B. All doses are allocated to the primary variant at each target, that is, we vary x while $y_{A}=y_{B}=1$. Because both vaccines offer equal protection against the initial strain, the choice of x only impacts subsequent waves, to which conventional vaccination provides no protection (under our assumption of limited cross‐immunity). As a result, mosaic vaccination (0<x<1) is at least as good as conventional vaccination in terms of maximizing $E[S_{\text{final}}]$. In fact, when antigenic evolution either can cause secondary waves (case II, Fig. 3A) or is necessary for an initial wave (case III, Fig. 3B), any variation in targets across vaccine doses, 0<x<1, is superior to conventional vaccination in terms of maximizing the expected number of individuals who do not contract the disease.

**Figure 4:**
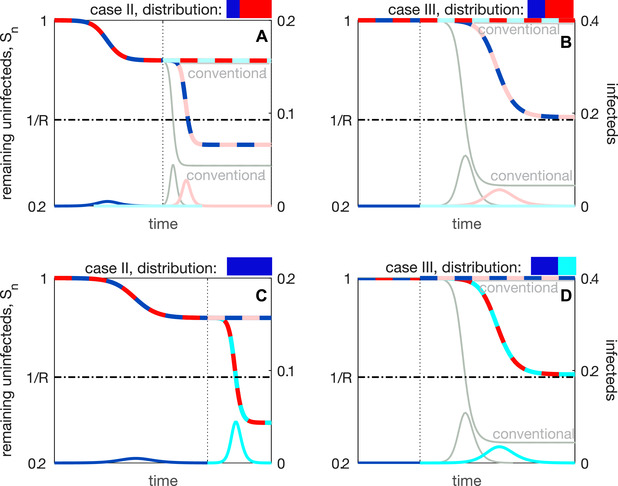
Epidemiological dynamics when optimally distributing across targets (A and B) or variants (C and D) when vaccine escape is rare. In all panels, there is a small probability of a single mutation at either target, giving rise to two equally probable sequences, $A_{0}B_{0}\to A_{1}B_{0}$ and $A_{0}B_{0}\to A_{0}B_{1}$; both of these sequences are shown. For the latter sequence, conventional vaccination blocks the second epidemic wave and so matches with the horizontal multi‐colored line in each panel. For the former sequence, conventional vaccination performs poorly, and is shown in light gray, except in (C) when the optimal distribution is also conventional vaccination. In Figures S1 and S2, we show the optimal distribution and the performance metrics for mosaic vaccination when vaccine escape is rare.

Perhaps more surprisingly, Figure 3C demonstrates that for cases II and III, an equal distribution of doses between targets, x=1/2, is rarely the best strategy for maximizing $E[S_{\text{final}}]$, regardless of the likelihood of vaccine escape. This is because there are two equally probable sequences of antigenic change, differentiated by which target changes first (i.e., is the second wave caused by $A_{1}B_{0}$ or $A_{0}B_{1}$?). We can choose x to maximize vaccine protection against either one of these sequences. If vaccine escape is common, this means choosing x so that for one of the sequences, $S_{\text{final}}=1/R$ as shown in Figure 3A and B. If vaccine escape is rare, this means choosing x to block the second wave of one sequence, while allocating the excess doses against the other target (Fig. 4A and B). In comparison with x=1/2, either of these choices of $x^{*}$ will reduce protection against the other sequence. However, for a wide range of parameter space, Figure 3D demonstrates that the average outcome of $x^{*}$ across the two possible sequences outperforms the expected outcome when x=1/2, and always outperforms conventional vaccination (Fig. 3C, and D; Fig. S1C and   D).

The advantage of distributing across targets over conventional vaccination is not limited to increasing the remaining uninfected, $E[S_{\text{final}}]$. Figure 3E and F show that distributing across targets also increases vaccine efficacy and vaccine matching; results that also hold when vaccine escape is rare (Fig. S1E and   F). Thus not only does mosaic vaccination improve population level outcomes, it also reduces individual‐level risks associated with weaker protection against symptoms from mismatched vaccines.

### VACCINES DISTRIBUTED ACROSS VARIANTS

Next, we consider distributing equally effective vaccines between two variants of target A, that is, setting x=1 and varying $y_{A}$. Clearly, any $y_{A}<1$ will reduce the protection against the initial strain, and so if vaccine coverage is sufficiently weak (case I), all doses should be allocated to the primary variant, $y_{A}=1$ (conventional vaccination). In cases II and III, however, conventional vaccination performs poorly because the population is effectively unvaccinated against any variation at target A (Fig. 2B and C).

If vaccine escape is rare, then it is optimal to maximize protection against the primary variant, $A_{0}$. In case II, because conventional vaccination is not sufficiently strong to block the initial $A_{0}B_{0}$ epidemic wave, all doses should be allocated against $A_{0}$ ($y_{A}=1$; Fig. 4C). In case III, because conventional vaccination is sufficiently strong to block the $A_{0}B_{0}$ epidemic wave, $y_{A}$ should be chosen such that the reproductive number for strain $A_{0}B_{0}$ is reduced to one. Here, any excess doses not needed to block the $A_{0}B_{0}$ epidemic wave are diverted to protect against the (rare) possibility that a strain carrying the variant $A_{1}$ emerges (Fig. 4D, Fig. S2).

**Figure 5:**
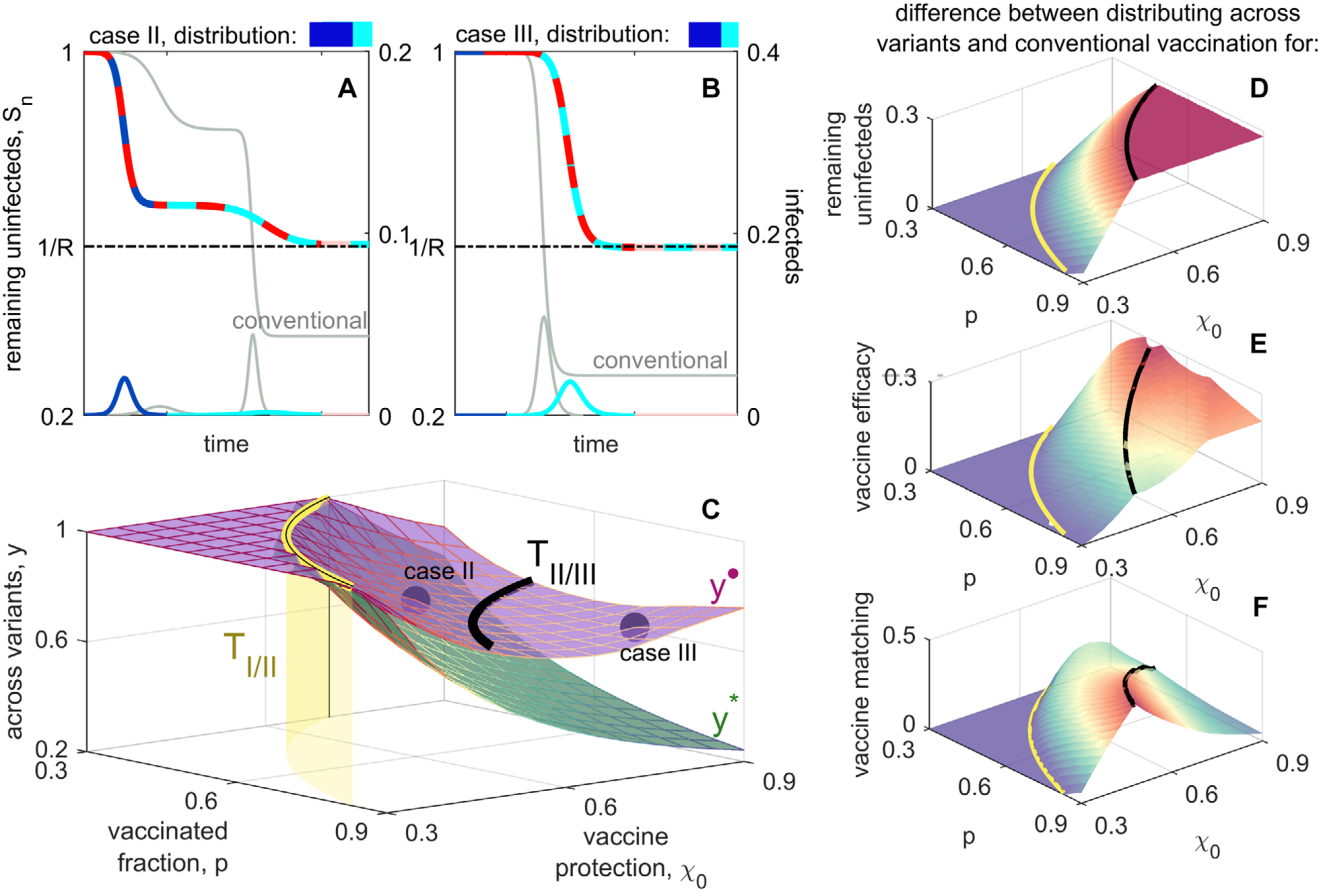
Optimal distribution of vaccines across variants when vaccine escape is common. (A) and (B) Example epidemic dynamics for case II (A) and case III (B), with conventional vaccination (light gray) shown for reference. Values of p, $\chi_{0}$, and the optimal distribution $y^{•}$ for these plots are indicated by the purple circles in (C). For both panels, there is only one sequence of interest, corresponding to antigenic changes at target A: $A_{0}B_{0}\to A_{1}B_{0}\to A_{2}B_{0}$. In case II, the optimal distribution weakens protection against the first epidemic wave, as compared to conventional vaccination so as to provide better protection against the second; the outcome across both epidemics is substantially improved. In case III, the optimal distribution across variants is to block the epidemic wave by strain $A_{0}B_{0}$ and then allocate any excess doses against variant $A_{1}$. (C) The optimal distribution across variants, $y^{•}$ and $y^{*}$ as vaccine coverage, p, and protection, $\chi_{0}$, vary. In (D)–(F), it is shown how distributing across variants (using $y^{•}$) outperforms conventional vaccination for three metrics; each panel shows the difference between distributing across targets and conventional vaccination. (D) The difference in remaining uninfected, $E[S_{\text{final}}]$. (E) The difference in vaccine efficacy, measured as attack rate over the course of the epidemic. (F) The difference in vaccine matching, defined as the probability that an individual that is both vaccinated and infected is infected by a strain that they were vaccinated against. In all panels, R=1.75 and the thresholds $T_{\frac{I}{II}}\left(p\right)$ (yellow surface) and $T_{\frac{II}{III}}\left(p\right)$ (black lines) are included for reference.

When vaccine escape is common, we can always choose $y_{A}$ to ensure that $E[S_{\text{final}}]=1/R$, as illustrated in Fig. 5A and B. Thus in contrast to distributing vaccine doses between targets, when distributing across variants we can, in theory, always attain the optimum. This can be achieved with two values of $y_{A}$: we can use strategy $y^{*}$, such that the population reaches herd immunity after the first wave; or we can use strategy $y^{•}$, such that herd immunity is reached after the second wave (Fig. 5C). Although both $y^{*}$ and $y^{•}$ maximize $E[S_{\text{final}}]$, from a public health perspective $y^{•}$ is the superior option as it maximizes $E[S_{\text{final}}]$ after at least one antigenic change, and so two potential waves, rather than one epidemic and no antigenic change. By staggering the waves, the peak burden on the healthcare system is reduced (e.g., Qualls et al. (2017)). Indeed, the less desirable outcome of $y^{*}$ could simply be achieved using conventional vaccination with lower coverage. In Figure 5A and B, we show example epidemiological dynamics for $y^{•}$.

As was the case when distributing across targets, the advantage of distributing across variants (using $y^{•}$) over conventional vaccination is not limited to increasing the remaining uninfecteds, $E[S_{\text{final}}]$ (Fig. 5D). Distributing across variants also increases vaccine efficacy (Fig. 5E) and vaccine matching (Fig. 5F). In case III, these results also hold when vaccine escape is rare (Fig. S2E and   F). Thus distributing across variants improves both population level outcomes as well as reducing individual risk.

### DISTRIBUTING ACROSS TARGETS AND VARIANTS SIMULTANEOUSLY

Finally, consider distributing vaccines across both targets and variants. When we are free to choose all three of $\left(x,y_{A},y_{B}\right)$, many different combinations can lead to similar $S_{n}$, so we can apply further constraints to identify optimal combinations. We opt to impose the constraint that $y_{A}=y_{B}=y$; this allows us to isolate whether distributing over targets or variants is more important.

Under these circumstances, the outcome is as we would expect from consideration of our previous results. When the likelihood of vaccine escape is low, the optimal solution is to rely on distributing doses between targets (varying x) while targeting the primary variant, y=1. As vaccine escape becomes more common, the optimal strategy is to distribute between variants (varying y), while keeping doses equally divided across targets, x=1/2. The reason for this is intuitive: when there is a low probability of a second wave, we want to maintain maximum protection against the initial strain (y=1), but are free to distribute between targets so as to block one of the epidemic sequences. As the likelihood of vaccine escape increases, a wave by a secondary variant is increasingly likely, and so it can be beneficial to sacrifice protection against the initial strain to allocate doses against the secondary variants. Although we previously showed x=1/2 is not optimal when distributing across targets only, if we are free to distribute across both variants and targets, and are allocating doses to both the primary and secondary variant, the logic changes. Specifically, as either epidemic sequence is equally likely, when relying on distributing between variants it makes sense to distribute equally between targets (x=1/2; Fig. S3).

### MORE COMPLEX EPIDEMIOLOGICAL SCENARIOS

In the previous sections, we examined an idealized epidemiological model in order to clearly lay out mosaic vaccination as a proof‐of‐concept. Here we briefly consider how incorporating different epidemiological assumptions affects our predictions; to do so, we retain our focus on cases II and III, and discuss two reasons why mosaic vaccination can be more effective than conventional vaccination. More details on the calculations used in this section can be found in the Supporting Information S10.

#### Conventional vaccination leads to overshoot

By definition, conventional vaccination allocates the greatest possible protection against strains carrying variant $A_{0}$, and so following the first epidemic wave, the largest possible pool of individuals with weak or no protection against variant $A_{1}$ remains. As a result, if strain $A_{1}B_{j}$ enters the population, it will cause the largest possible epidemic wave. Moderately increasing the size of the first wave produces a disproportionate reduction in the size of the second wave by increasing the fraction of individuals with naturally acquired immunity to variant $A_{1}$. This will improve the outcome over the course of the epidemic (Restif and Grenfell 2007; Zarnitsyna et al. 2018).

That reducing protection against the first wave can be beneficial has clear implications for mosaic vaccination. In particular, two of the seemingly largest challenges to mosaic vaccination would be accurately predicting future variants and/or identifying two roughly equally effective targets. However, as increasing the size of the first wave can reduce total infections, even if the vaccine doses allocated toward either future variants or against target B generate a substantially inferior immune response to those doses allocated toward current variants or against target A (e.g., due to inaccurate predictions or unequal targets), mosaic vaccination still does no worse than conventional vaccination.

#### Mosaic vaccination provides greater protection against future epidemic waves

In general, however, mosaic vaccination does not simply involve “wasting” doses. By allocating doses against the other target or against other variants, then all else being equal, it provides greater protection against future epidemic waves. Thus if our model were extended to include epidemiological factors that increase the size of future waves, we should still expect mosaic vaccination to generally outperform conventional vaccination.

There are two relevant examples. First, suppose there are two possible epidemic waves, and some fraction of recovered individuals lose their naturally acquired immunity between waves; for example, influenza infection triggers a short‐term, broadly neutralizing immune response that decays over time (Kucharski et al. 2018). This will increase the availability of susceptible hosts following the first wave, making the second wave worse than it would be otherwise, and so mosaic vaccination will still generally outperform conventional vaccination. Second, in addition to undergoing antigenic evolution, the pathogen may also undergo life‐history evolution, for example, by becoming more transmissible following the first wave. As this will worsen future epidemic waves, again mosaic vaccination will generally outperform conventional vaccination. Only if the pathogen becomes significantly less transmissible, and we are distributing across variants, will mosaic vaccination be outperformed by conventional vaccination; here the doses allocated to the future variants are “wasted,” but additionally, the second epidemic wave is significantly smaller than expected as the number of infections required to reach herd immunity decreases.

## Discussion

We have used a simple model to explore the optimal distribution of vaccines that differ in antigenic targets and/or specific variants at those targets (i.e., strains). We show that with weak vaccines (either low coverage or weak protection), evolutionary change in the pathogen population does not alter epidemiological outcomes and so a mosaic vaccination strategy, in which individuals receive one of a set of possible vaccines, is not better than a conventional strategy. In cases where pathogen evolution can lead to successive waves of an epidemic, or is required for an initial wave, using a mosaic vaccination strategy often leads to better outcomes than giving all individuals identical vaccines.

Vaccines distributed across targets are equally effective against the initial circulating strain, thus producing outcomes at least as good as conventional vaccination. However, if antigenic evolution occurs, the benefits of mosaic vaccination are realized. Although a conventional vaccine would offer no protection against a strain that had evolved at one of the two targets (given our assumptions about cross‐immunity), some protection would be maintained in a population that received a mosaic strategy. Perhaps surprisingly, the optimal distribution of vaccine doses between targets is not 50:50—better outcomes can be achieved on average with unequal allocation by providing greater protection against one sequence of antigenic changes, while still retaining some protection against the other possible sequence (unlike conventional vaccination).

While neither our focus nor motivation, it is interesting to consider our results in the context of the ongoing COVID‐19 pandemic, given the rapid and parallel development of dozens of vaccines against SARS‐CoV‐2 (Al‐Kassmy et al. 2020; Poland et al. 2020; World Health Organization 2020). Although many COVID‐19 vaccines in development target the spike protein, some target epitopes in other proteins (Al‐Kassmy et al. 2020) and the potential for multi‐epitope (i.e., cocktail) vaccines are being investigated (e.g., Bhattacharya et al. 2020; Enayatkhani et al. 2021; Kar et al. 2020; Kalita et al. 2020). If in the future multiple COVID‐19 vaccines were approved with similar efficacies (i.e., roughly equal protection against the predominant circulating strain), then our results suggest that to minimize the risk of evolutionary escape and reduce the total number of individuals who get infected, the vaccines should be used in a mosaic strategy (*sensu* REX Consortium 2013). Multiple plausible vaccine targets exist for other infectious diseases (e.g., hemagglutinin and neuraminidase in influenza; Johansson and Cox 2011); our model provides a framework for evaluating the efficacy of mosaic vaccination strategies given the details of specific diseases.

What happens when vaccines are distributed across variants of a given target is perhaps more subtle. If evolutionary change in antigens is unlikely, then diverting any vaccine doses away from the predominant strain can lead to worse epidemiological outcomes. However, even if antigenic change is unlikely, if vaccine efficacy ($\chi_{0}$) is sufficiently strong or coverage (p) is sufficiently high (i.e., our case III), then from a population perspective better outcomes can be achieved by vaccinating a fraction of the population against a secondary variant. As the likelihood of waves from antigenically distinct strains increases, then a trade‐off emerges between protection in an initial wave versus subsequent ones. Put simply, a small initial wave due to strong vaccine protection against an initial variant leaves a large pool of susceptible individuals that could be exploited by a subsequent strain (assuming weak cross‐immunity from vaccination), while a large initial wave due to poor vaccine protection leaves few individuals susceptible to future waves (assuming strong cross‐immunity from natural infections). The optimal strategy essentially titrates between these scenarios, offering intermediate protection against initial and subsequent strains and dampening—but not eliminating—individual waves. This intermediate strength optimal strategy echoes results from previous work: in an explicit two‐strain epidemiological model, if vaccination disproportionately impacts one strain (and vaccine‐induced immunity is weaker than natural immunity), then increasing vaccine coverage or protection can lead to outgrowth of infections with the second strain and worse outcomes overall (Restif and Grenfell 2007; Zarnitsyna et al. 2018).

Distributing across variants seems intuitively risky. For influenza, for example, substantial work goes into choosing which variant of each flu subtype will be included in a vaccine in a given year (e.g., Morris et al. 2018). Part of the decision is based on the frequency of circulating variants and the likelihood that any one variant will seed the coming year's seasonal epidemic. If there is good reason to expect a particular variant will circulate predominantly, it would seem unethical to allocate any vaccine doses to a different variant. Yet our results show that over a considerable range of parameters (i.e., vaccine efficacy and coverage, likelihood of antigenic change), the conventional strategy does not give rise to the best outcomes and so the principle of equipoise would not be breached by distributing vaccines across variants. For influenza, evolutionary predictions are improving (e.g., Łuksza and Lässig 2014; Neher et al. 2016; Morris et al. 2018); our results suggest a novel way of using those predictions for vaccine design, assuming any practical barriers can be overcome.

Somewhat akin to mosaic vaccination, previous work has explored the epidemiological and evolutionary consequences of vaccines that produce variable effects across hosts. First, using a data‐driven model of a viral disease of fish, Langwig et al. (2017) showed that vaccines that induce variation in susceptibility across hosts can lead to better epidemiological outcomes than vaccines that have individually invariant effects (analogous to theoretical results exploring the effects of natural variation in susceptibility (Dwyer et al. 2000; Lloyd‐Smith et al. 2005; Lively 2010). This is because with variation, average susceptibility declines over the course of the epidemic, as hosts that are the most susceptible get infected earlier on average. Likewise, in our model, distributing vaccine doses across targets or variants changes the landscape of host susceptibility both initially and as circulating pathogens evolve. In our case, distributing across targets mitigates the increase in susceptibility that would occur with conventional vaccines following antigenic evolution, while distributing across variants can actually reverse it. Second, in seeking to explain why few vaccines have failed in the face of pathogen evolution relative to the alarming rise and spread of drug resistance, Kennedy and Read (2017) suggest a potential role for individual variation in response to vaccination. Much of that paper is focused on processes that impede the evolutionary emergence of resistance or escape within a host, and vaccines with the potential to generate immunity against multiple antigens may lead to more diverse within‐host selection pressures on (and thus greater evolutionary hurdles for) pathogens compared to the use of a single drug. But Kennedy and Read (2017) also note that if the repertoire of vaccine‐induced immune responses varies across individuals, then that rugged fitness landscape plays out at the host population level too, and the population may be more robust to the introduction of a new pathogen variant. In practice, for influenza at least, there is strong evidence that individuals do respond qualitatively differently to the same vaccine, due to differences in past history of exposure (e.g., Gostic et al. 2016; reviewed in Cobey and Hensley 2017). Our work reinforces the idea that, in some cases, this variation can be beneficial from a public health perspective (Kennedy and Read 2017) and so generating such variation could be an explicit aim of vaccination.

Our model makes a number of key assumptions. First, antigenic change was assumed to arise without consideration of its source. This is reasonable if antigenic novelty originates outside the focal population. For example, it may come from a reservoir animal population or be otherwise imported from a source population (e.g., influenza A; Bedford et al. 2015; Russell et al. 2008). If instead we focused strictly on antigenic change from de novo mutation in the focal population, then unless mutations are very likely, conventional vaccination tends to perform better than our results show; in this case, it is typically better to “hit hard” in the hopes of preventing antigenic change. Second, we assumed cross‐protection was limited. The “broadness” of vaccine cross‐immunity determines the reduction in vaccine protection against new strains. If vaccine cross‐immunity is broad, antigenic change will cause a small reduction in vaccine protection, and so a smaller subsequent wave. Thus increasing cross‐protection increases the efficacy of conventional vaccination in cases II and III. In our model, the broadness of cross‐immunity will ultimately depend on the units of antigenic space and the time scale of interest. Third, we assumed each wave consists of a single strain. Although this may be reasonable if antigenic variation originates elsewhere, it is less likely when variation is generated by de novo mutation. If our results were extended to include multi‐strain waves, the predicted efficacy of conventional vaccination would be weakened, as whenever an escape mutant arises during an ongoing wave, there are more individuals without infection‐acquired immunity and so susceptible to the escape mutant. This would lead to a larger wave by the strain with limited vaccine protection.

Although the evolutionary and epidemiological consequences of universal vaccines have received some theoretical attention (e.g., Subramanian et al. 2016), here we explored the potential for vaccination strategies to essentially generate universal coverage at the host population level by delivering variable vaccines to individuals. This mosaic strategy generates heterogeneity in the host population (and, thus, the fitness landscape for pathogens), which has long been thought to be protective against disease outbreaks (Elton 1958). Theory predicts that “naturally” variable host populations are less likely to experience sustained disease spread (e.g., Lloyd‐Smith et al. 2005; Lively 2010), and the protective effect of host variation has been empirically demonstrated in a number of experimental (e.g., Altermatt and Ebert 2008; Common et al. 2020) and natural systems (Ekroth et al. 2019), especially those in which rapid host evolution is unlikely (Gibson and Nguyen 2020). Our work suggests that vaccination strategies that harness—and, in fact, generate—variation can often outperform conventional vaccines.

## AUTHOR CONTRIBUTION

DVM, NM and LMW developed the research question. DVM designed the model with input from LMW and NM, and performed the mathematical analysis. DVM, LMW and NM wrote the manuscript. LMW and NM contributed equally.

## Supporting information


**Figure S1**: Optimal distribution of vaccines across targets when vaccine escape is rare.
**Figure S2**: Optimal distribution of vaccines across variants when vaccine escape is rare.
**Figure S3**: Optimal distribution of vaccines when distributing between targets (panel **a**) and variants (panel **b**) simultaneously.
**Figure S4**: Effect of waning natural immunity.
**Figure S5**: Outcome when life‐history evolution occurs simultaneously with antigenic evolution.
**Figure S6**: Peak protection vs broad cross‐protection.
**Figure S7**: Mosaic vaccination can still outperform conventional vaccination when targets provide unequal protection or protection against variants is reduced.

Supporting Information

## References

[evl3252-bib-0001] Al‐Kassmy, J. , J. Pedersen , and G. Kobinger . 2020. Vaccine candidates against coronavirus infections. Where does COVID‐19 stand? Viruses 12:861. 10.3390/v12080861 PMC7472384 32784685

[evl3252-bib-0002] Altermatt, F. , and D. Ebert . 2008. Genetic diversity of *Daphnia magna* populations enhances resistance to parasites. Ecol. Lett. 11:918–928. 18479453 10.1111/j.1461-0248.2008.01203.x

[evl3252-bib-0003] Bedford, T. , S. Riley , I. Barr , S. Broor , M. Chadha , N. Cox , et al. 2015. Global circulation patterns of seasonal influenza viruses vary with antigenic drift. Nature 532:217–220. 10.1038/nature14460 PMC4499780 26053121

[evl3252-bib-0004] Bhattacharya, M. , A. R. Sharma , P. Patra , P. Ghosh , G. Sharma , B. C. Patra , et al. 2020. Development of epitope‐based peptide vaccine against novel coronavirus 2019 (SARS‐COV‐2): Immunoinformatics approach. J. Med. Virol. 92:618–631. 32108359 10.1002/jmv.25736 PMC7228377

[evl3252-bib-0005] Chen, Y. , T. J. Wohlbold , N. Zheng , M. Huang , Y. Huang , K. E. Neu , et al. 2018. Influenza infection in humans induces broadly cross‐reactive and protective neuraminidase‐reactive antibodies. Cell 173:417–429. 29625056 10.1016/j.cell.2018.03.030 PMC5890936

[evl3252-bib-0006] Cobey, S. , and S. E. Hensley . 2017. Immune history and influenza virus susceptibility. Curr. Opin. Virol. 22:105–111. 28088686 10.1016/j.coviro.2016.12.004 PMC5467731

[evl3252-bib-0007] Common, J. , D. Walker‐ünderhauf , S. Houte , and E. R. Westra . 2020. Diversity in CRISPR‐based immunity protects susceptible genotypes by restricting phage spread and evolution. J. Evol. Biol. 33:1097–1108. 10.1111/jeb.13638 32383796

[evl3252-bib-0008] Dwyer, G. , J. Dushoff , J. S. Elkington , and S. A. Levin . 2000. Pathogen‐driven outbreaks in forest defoliators revisited: building models from experimental data. Am. Nat. 156:105–120. 10856195 10.1086/303379

[evl3252-bib-0009] Ekroth, A. K. E. , C. Rafaluk‐Mohr , and K. C. King . 2019. Host genetic diversity limits parasite success beyond agricultural systems: a meta‐analysis. Proc. R. Soc. B 286:20191811. 10.1098/rspb.2019.1811 PMC6774778 31551053

[evl3252-bib-0010] Elton, C. S. 1958. The ecology of invasions by animals and plants. John Wiley, New York, NY.

[evl3252-bib-0011] Enayatkhani, M. , M. Hasaniazad , S. Faezi , H. Gouklani , P. Davoodian , N. Ahmadi , et al. 2021. Reverse vaccinology approach to design a novel multi‐epitope vaccine candidate against COVID‐19: an *in silico* study. J. Biomol. Struct. Dyn. 39:2857–2872. 32295479 10.1080/07391102.2020.1756411 PMC7196925

[evl3252-bib-0012] Fiers, W. , M. De Filette , K. E. Bakkouri , B. Schepens , K. Roose , M. Schotsaert , et al. 2009. M2e‐based universal influenza A vaccine. Vaccine 27:6280–6283. 19840661 10.1016/j.vaccine.2009.07.007

[evl3252-bib-0013] Gibson, A. K. , and A. E. Nguyen . 2020. Does genetic diversity protect host populations from parasites? A meta‐analysis across natural and agricultural systems. Evol. Lett. 5:16–32. 33552533 10.1002/evl3.206 PMC7857278

[evl3252-bib-0014] Gostic, K. M. , M. Ambrose , M. Worobey , and J. O. Lloyd‐Smith . 2016. Potent protection against H5N1 and H7N9 influenza via childhood hemagglutinin imprinting. Science 354:722–726. 27846599 10.1126/science.aag1322 PMC5134739

[evl3252-bib-0015] Greaney, A. J. , A. N. Loes , L. E. Gentles , K. H. D. Crawford , T. N. Starr & K. D. Malone et al. 2021. The SARS‐CoV‐2 mRNA‐1273 vaccine elicits more RBD‐focused neutralization, but with broader antibody binding within the RBD. bioRxiv.

[evl3252-bib-0016] Handel, A. , I. Longini , and R. Antia . 2007. What is the best control strategy for multiple infectious disease outbreaks? Proc. R. Soc. B 274:833–837. 10.1098/rspb.2006.0015 PMC2093965 17251095

[evl3252-bib-0017] Hausdorff, W. P. , and W. P. Hanage . 2016. Interim results of an ecological experiment — conjugate vaccination against the pneumococcus and serotype replacement. Hum. Vaccin. Immunother. 12:358–374. 26905681 10.1080/21645515.2015.1118593 PMC5049718

[evl3252-bib-0018] Johansson, B. E. , and M. M. J. Cox . 2011. Influenza viral neuraminidase: the forgotten antigen. Expert Rev. Vaccines 10:1683–1695. 22085172 10.1586/erv.11.130

[evl3252-bib-0019] Kalita, P. , A. K. Padhi , K. Y. Zhang , and T. Tripathi . 2020. Design of a peptide‐based subunit vaccine against novel coronavirus SARS‐CoV‐2. Microb. Pathog. 145:104236. 32376359 10.1016/j.micpath.2020.104236 PMC7196559

[evl3252-bib-0020] Kar, T. , U. Narsaria , S. Basak , D. Deb , F. Castiglione , D. M. Mueller , et al. 2020. A candidate multi‐epitope vaccine against SARS‐CoV‐2. Sci. Rep. 10:10895. 32616763 10.1038/s41598-020-67749-1 PMC7331818

[evl3252-bib-0021] Kennedy, D. A. , and A. F. Read . 2017. Why does drug resistance readily evolve but vaccine resistance does not? Proc. R. Soc. B 284:20162562. 10.1098/rspb.2016.2562 PMC5378080 28356449

[evl3252-bib-0022] Kennedy, D. A. , and A. F. Read 2018. Why the evolution of vaccine resistance is less of a concern than the evolution of drug resistance. Proc. Natl. Acad. Sci. 115:12878–12886. 30559199 10.1073/pnas.1717159115 PMC6304978

[evl3252-bib-0023] Kim, J. H. , J. Liepkalns , A. J. Reber , X. Lu , N. Music , J. Jacob , et al. 2016. Prior infection with influenza virus but not vaccination leaves a long‐term immunological imprint that intensifies the protective efficacy of antigenically drifted vaccine strains. Vaccine 34:495–502. 26706277 10.1016/j.vaccine.2015.11.077 PMC4713344

[evl3252-bib-0024] Kucharski, A. , J. Lessler , D. A. T. Cummings , and S. Riley , 2018. Timescales of influenza A/H3N2 antibody dynamics. PLoS Biol. 16:e2004974. 30125272 10.1371/journal.pbio.2004974 PMC6117086

[evl3252-bib-0025] Langwig, K. , A. Wargo , D. Jones , J. Viss , B. Rutan , N. Egan , et al. 2017. Vaccine effects on heterogeneity in susceptibility and implications for population health management. mBio 8:e00796–17. 29162706 10.1128/mBio.00796-17 PMC5698548

[evl3252-bib-0026] Lin, J. , V. Andreasen , R. Casagrandi , and S. Levin , 2003. Traveling waves in a model of influenza A drift. J. Theor. Biol. 222:437–445. 12781742 10.1016/s0022-5193(03)00056-0

[evl3252-bib-0027] Lively, C. M. , 2010. The effect of host genetic diversity on disease spread. Am. Nat. 175:E149–E152. 20388005 10.1086/652430

[evl3252-bib-0028] Lloyd‐Smith, J. O. , S. J. Schreiber , P. E. Kopp , and W. M. Getz , 2005. Superspreading and the effect of individual variation on disease emergence. Nature 438:355–359. 16292310 10.1038/nature04153 PMC7094981

[evl3252-bib-0029] Łuksza, M. , and M. Lässig , 2014. A predictive fitness model for influenza. Nature 507:57–61. 24572367 10.1038/nature13087

[evl3252-bib-0030] Mallajosyula, V. V. A. , M. Citron , F. Ferrara , X. Lu , C. Callahan , G. J. Heidecker , et al. 2014. Influenza hemagglutinin stem‐fragment immunogen elicits broadly neutralizing antibodies and confers heterologous protection. Proc. Natl. Acad. Sci. 111:E2514–E2523. 24927560 10.1073/pnas.1402766111 PMC4078824

[evl3252-bib-0031] MATLAB . 2019. Ver 9.6.0 (R2019a). The MathWorks Inc., Natick, MA.

[evl3252-bib-0032] Morris, D. H. , K. M. Gostic , S. Pompei , T. Bedford , M. Łuksza , R. A. Neher , et al. 2018. Predictive modeling of influenza shows the promise of applied evolutionary biology. Trends Microbiol. 26:102–118. 29097090 10.1016/j.tim.2017.09.004 PMC5830126

[evl3252-bib-0033] Nachbagauer, R. , and F. Krammer . 2017. Universal influenza virus vaccines and therapeutic antibodies. Clin. Microbiol. Infect. 23:222–228. 28216325 10.1016/j.cmi.2017.02.009 PMC5389886

[evl3252-bib-0034] Neher, R. A. , T. Bedford , R. S. Daniels , C. A. Russell , and B. I. Shraiman . 2016. Predictive, dynamics, and visualization of antigenic phenotypes of seasonal influenza viruses. Proc. Natl. Acad. Sci. 113:E1701–E1709. 26951657 10.1073/pnas.1525578113 PMC4812706

[evl3252-bib-0035] Okuno, Y. , Y. Isegawa , F. Sasao , and S. Ueda . 1993. A common neutralizing epitope conserved between the hemagglutinins of influenza A virus H1 and H2 strains. J. Virol. 67:2552–2558. 7682624 10.1128/jvi.67.5.2552-2558.1993 PMC237575

[evl3252-bib-0036] Petrova, V. N. , and C. A. Russell . 2018. The evolution of seasonal influenza viruses. Nat. Rev. Microbiol. 16:47–60. 29081496 10.1038/nrmicro.2017.118

[evl3252-bib-0037] Poland, G. A. , I. G. Ovsyannikova , S. N. Crooke , and R. B. Kennedy . 2020. SARS‐CoV‐2 vaccine development: current status. Mayo Clin. Proc. 95:2172–2188. 33012348 10.1016/j.mayocp.2020.07.021 PMC7392072

[evl3252-bib-0038] Qualls, N. , A. Levitt , N. Kanade , N. Wright‐Jegede , S. Dopson , M. Biggerstaff , et al. 2017. Community mitigation guidelines to prevent pandemic influenza ‐ United States. MMWR. Recomm. Rep. 66:11–34. 10.15585/mmwr.rr6601a1 PMC5837128 28426646

[evl3252-bib-0039] Restif, O. , and B. T. Grenfell . 2007. Vaccination and the dynamics of immune evasion. J. R. Soc. Interface 4:143–153. 17210532 10.1098/rsif.2006.0167 PMC2358969

[evl3252-bib-0040] REX Consortium . 2013. Heterogeneity of selection and the evolution of resistance. Trends Ecol. Evol. 28:110–118. 23040463 10.1016/j.tree.2012.09.001

[evl3252-bib-0041] Russell, C. A. , T. C. Jones , I. G. Barr , N. J. Cox , R. Garten , V. Gregory , et al. 2008. The global circulation of seasonal influenza A (H3N2) viruses. Science 320:340–346. 18420927 10.1126/science.1154137

[evl3252-bib-0042] Smith, D. J. , A. S. Lapedes , J. C. de Jong , T. M. Bestebroer , G. F. Rimmelzwaan , A. D. M. E. Osterhaus , et al. 2004. Mapping the antigenic and genetic evolution of influenza virus. Science 305:371–376. 15218094 10.1126/science.1097211

[evl3252-bib-0043] Subramanian, R. , A. L. Graham , B. T. Grenfell , and N. Arinaminpathy . 2016. Universal or specific? A modeling‐based comparison of broad‐spectrum influenza vaccines against conventional, strain‐matched vaccines. PLoS Comput. Biol. 12:e1005204. 27977667 10.1371/journal.pcbi.1005204 PMC5157952

[evl3252-bib-0044] Viboud, C. , K. Gostic , M. I. Nelson , G. E. Price , A. Perofsky , K. Sun , et al. 2020. Beyond clinical trials: evolutionary and epidemiological considerations for development of a universal influenza vaccine. PLOS Pathog. 16:e1008583. 32970783 10.1371/journal.ppat.1008583 PMC7514029

[evl3252-bib-0045] World Health Organization . 2017. Ten years in public health, 2007‐2017. Geneva. Report by Dr. M. Chan, Director‐General. Available at https://www.who.int/publications/10‐year‐review/en/.

[evl3252-bib-0046] World Health Organization 2020. Draft landscape of COVID‐19 candidate vaccines. Geneva. Available at https://www.who.int/publications/m/item/draft‐landscape‐of‐covid‐19‐candidate‐vaccines.

[evl3252-bib-0047] Xue, K. S. , and J. D. Bloo . 2020. Linking influenza virus evolution within and between human hosts. Virus Evol. 6:Veaa010. 32082616 10.1093/ve/veaa010 PMC7025719

[evl3252-bib-0048] Zarnitsyna, V. I. , I. Bulusheva , A. Handel , I. M. Longini , M. E. Halloran , and R. Antia . 2018. Intermediate levels of vaccination coverage may minimize seasonal influenza outbreaks. PLoS One 13:e0199674. 29944709 10.1371/journal.pone.0199674 PMC6019388

