# Supplementary material for: Mosaic vaccination: How distributing different vaccines across a population could improve epidemic control
Source: Evol Lett. 2021 Aug 23;5(5):458–71. doi: 10.1002/evl3.252 (PMC8484727; doi:10.1002/evl3.252)
Supplement: Supplementary file 1 — Figure S1: Optimal distribution of vaccines across targets when vaccine escape is rare. Figure S2: Optimal distribution of vaccines across variants when vaccine escape is rare. Figure S3: Optimal distribution of vaccines when distributing between targets (panel a) and variants (panel b) simultaneously. Figure S4: Effect of waning natural immunity. Figure S5: Outcome when life‐history evolution occurs simultaneously with antigenic evolution. Figure S6: Peak protection vs broad cross‐protection. Figure S7: Mosaic vaccination can still outperform conventional vaccination when targets provide unequal protection or protection against variants is reduced. [file EVL3-5-458-s002.pdf]

Supplementary Information for *Mosaic vaccination: how distributing different vaccines across a population could improve epidemic control*

David V. McLeod[1], Lindi M. Wahl*[2] and Nicole Mideo*[3]

[1]Centre D'Ecologie Fonctionnelle & Evolutive
CNRS, Montpellier, France
david.mcleod@cefe.cnrs.fr

[2]Department of Applied Mathematics,
Western University, London, Canada
lwahl@uwo.ca

[3]Department of Ecology and Evolutionary Biology
University of Toronto, Toronto, Canada
nicole.mideo@utoronto.ca

S1 Preliminaries

We consider a pathogen with two vaccine targets, A and B, each of which can exhibit antigenic variation. We let A_iB_j denote a pathogen strain with variant i at target A and variant j at target B. At each target, antigenic space is one dimensional, such that as i (or j) increases, the strain A_iB_j is further away from the reference strain, which we denote A_0B_0. Using this reference strain, we will refer to the variant indexed 0 as the 'primary' variant and the variant indexed 1 as the 'secondary' variant, e.g., strain A_1B_0 has the secondary variant for target A and primary variant for target B.

Each vaccine has efficacy χ_0 against its intended target/variant combination; for example, the probability that strain A_0B_1 infects individuals vaccinated against B_1 is reduced by a factor $1 - \chi_0$. More generally, a vaccine protecting against variant $j = 0, 1, ...$ of one target reduces the probability of infection by a strain with variant i of the same target by a factor $\chi_{|i-j|}$. Thus unlike in the main text, here we allow vaccines to confer cross-protection, where the degree of cross-protection depends only on the antigenic distance between variants at the vaccine target and not the variant at the other target. In contrast, infection-acquired immunity offers full protection against all strains, thus vaccine protection is inferior to infection-acquired immunity. All numeric results and figures were obtained and created in Matlab [1]. The code used to generate these figures has been uploaded as a supplemental file.

*equal contribution

S2 Single epidemic wave

To model the epidemiological dynamics of a single epidemic wave, we use a standard SIR model with a single strain, say $A_\ell B_m$, present. Let β be a rate constant reflecting the number of disease transmissions caused by a single infectious individual, per unit time, in a fully susceptible population. Infections have mean duration δ such that in the absence of vaccination, $R = \beta\delta$ reflects the basic reproductive ratio of this pathogen in a population where the density of susceptibles is normalized to 1.

Let $I_i(t)$ denote the density of infected individuals with strain $i = A_\ell B_m$ at time t. Let $U(t)$ denote the density of unvaccinated individuals in the population at time t, and $V^{\tau_v}(t)$ denote the density of individuals vaccinated against target τ and variant v ($\tau_v \in \{A_0, A_1, B_0, B_1\}$). Then the epidemiological dynamics are given by the system of ordinary differential equations

$$\begin{aligned}\frac{\mathrm{d}U}{\mathrm{d}t} &= -\beta U I_i \\ \frac{\mathrm{d}V^{\tau_v}}{\mathrm{d}t} &= -\beta(1-\chi_{|v-i_\tau|})V^{\tau_v} I_i, \quad \tau_v \in \{A_0, A_1, B_0, B_1\} \\ \frac{\mathrm{d}I_i}{\mathrm{d}t} &= \Big(\beta U + \beta \sum_{\tau_v}(1-\chi_{|v-i_\tau|})V^{\tau_v} - \frac{1}{\delta}\Big) I_i\end{aligned} \tag{S1}$$

where i_τ is the variant of strain $i = A_\ell B_m$ at target τ, i.e., $i_A = \ell$ and $i_B = m$, and so $|v - i_\tau|$ is the distance in antigenic space between the variant targeted by the vaccine, v, and the variant carried by strain i. From system (S1), the basic reproductive number of pathogen strain i is

$$\mathcal{R}_0 = R\Big(U(0) + \sum_{\tau_v}(1-\chi_{|v-i_\tau|})V^{\tau_v}(0)\Big), \tag{S2}$$

where $R \equiv \beta\delta$. It follows that if $\mathcal{R}_0 > 1$, then an initially rare strain i will cause an epidemic.

The density of individuals who have not been infected by time t is

$$S(t) = U(t) + \sum_{\tau_v} V^{\tau_v}(t). \tag{S3}$$

Our objective is to derive from system (S1) an expression for the remaining uninfected individuals, $S(t)$, after sufficient time has elapsed for the epidemic wave to conclude, that is, we are interested in $S(\infty)$. To do so, notice in system (S1) that U is a monotonically decreasing function of time. Therefore, we can rescale time by U, which will give the reduced system

$$\begin{aligned}\frac{\mathrm{d}V^{\tau_v}}{\mathrm{d}U} &= (1-\chi_{|v-i_\tau|})\frac{V^{\tau_v}}{U}, \\ \frac{\mathrm{d}I_i}{\mathrm{d}U} &= -1 - \sum_{\tau_v}(1-\chi_{|v-i_\tau|})\frac{V^{\tau_v}}{U} + \frac{1}{RU}.\end{aligned} \tag{S4}$$

From (S4), we can directly solve for V^{τ_v}, that is,

$$V^{\tau_v} = V^{\tau_v}(0)\left(\frac{U}{U(0)}\right)^{1-\chi_{|v-i_\tau|}}. \tag{S5}$$

Using this result, we are left with the differential equation

$$\frac{\mathrm{d}I_i}{\mathrm{d}U} = -1 - \sum_{\tau_v}\frac{(1-\chi_{|v-i_\tau|})V^{\tau_v}(0)}{U}\left(\frac{U}{U(0)}\right)^{1-\chi_{|v-i_\tau|}} + \frac{1}{RU}. \tag{S6}$$

Applying the condition that strain i is initially rare, that is, $I_i(0) \approx 0$, this has solution

$$I_i(U) = \frac{1}{R}\ln\left(\frac{U}{U(0)}\right) + U(0) - U + \sum_{\tau_v} V^{\tau_v}(0)\left(1 - \left(\frac{U}{U(0)}\right)^{1-\chi_{|v-i_\tau|}}\right). \tag{S7}$$

As $t \to \infty$, we know that $I_i \to 0$, that is, the wave of strain i will eventually burn itself out of the host population. Therefore $U(\infty)$ is the solution of

$$0 = \frac{1}{R}\ln\left(\frac{U(\infty)}{U(0)}\right) + U(0) - U(\infty) + \sum_{\tau_v} V^{\tau_v}(0)\left(1 - \left(\frac{U(\infty)}{U(0)}\right)^{1-\chi_{|v-i_\tau|}}\right), \tag{S8}$$

which can be written, using equation (S5), as

$$0 = \frac{1}{R}\ln\left(\frac{U(\infty)}{U(0)}\right) + U(0) - U(\infty) + \sum_{\tau_v}\left(V^{\tau_v}(0) - V^{\tau_v}(\infty)\right). \tag{S9}$$

Therefore, there are two possibilities: if $\mathcal{R}_0 \leq 1$, $U(\infty) = U(0)$, otherwise $U(\infty)$ is the solution of equation (S9) satisfying $0 < U(\infty) < U(0)$. Once we have $U(\infty)$, we can then substitute it into equation (S3) to obtain $S(\infty)$.

S3 Successive epidemic waves

We now extend equation (S9) to consider a sequence of successive epidemic waves starting from the initial strain A_0B_0. To do so, let U_n and $V_n^{\tau_v}$ denote the density of susceptible, unvaccinated individuals and susceptible individuals vaccinated against τ_v, following the nth epidemic wave. This epidemic wave will have been due to a strain $n-1$ antigenic changes from the initial strain. Before any epidemic waves (or antigenic changes), i.e., $n = 0$, we have S_0 susceptible individuals without infection-acquired immunity. We vaccinate a fraction p of these. Of the vaccinated individuals, a fraction x vaccine doses are directed towards target A and $1-x$ directed towards target B. A fraction y_A of the doses for target A are used against the primary variant, A_0, while the remaining $1-y_A$ are used against the secondary variant, A_1; likewise, a fraction y_B of target B doses are used against B_0, while the remaining $1-y_B$ are used against the secondary variant, B_1. Therefore we initially have a density of $U_0 = S_0(1-p)$ unvaccinated, susceptible individuals, while the initial density of individuals vaccinated against each target and variant combination is:

$$V_0^{A_0} = S_0pxy_A, \qquad V_0^{A_1} = S_0px(1-y_A),$$
$$V_0^{B_0} = S_0p(1-x)y_B, \qquad V_0^{B_1} = S_0p(1-x)(1-y_B).$$

After the nth epidemic wave, the next pathogen strain, $A_\ell B_m$, will have undergone $n = \ell + m$ antigenic changes. There are 2^n possible antigenic sequences and $n+1$ possible $A_\ell B_m$ strains. For a given sequence of antigenic changes culminating in strain $A_\ell B_m$ following the nth epidemic wave, the basic reproductive number of strain $A_\ell B_m$ is

$$\mathcal{R}_0^{(n+1)} = R\big(U_n + (1-\chi_\ell)V_n^{A_0} + (1-\chi_{|\ell-1|})V_n^{A_1} + (1-\chi_m)V_n^{B_0} + (1-\chi_{|m-1|})V_n^{B_1}\big), \tag{S10}$$

that is, $\mathcal{R}_0^{(n+1)}$ indicates whether or not the $(n+1)^{\text{th}}$ epidemic wave will occur. Using the sequence $A_0B_0 \to A_0B_1 \to A_1B_1$ as an example, the basic reproductive number of strain A_1B_1 is:

$$\mathcal{R}_0^{(3)} = R\Big[U_2 + (1-\chi_1)V_2^{A_0} + (1-\chi_0)V_2^{A_1} + (1-\chi_1)V_2^{B_0} + (1-\chi_0)V_2^{B_1}\Big]. \tag{S11}$$

Note that, in general, U_n and $V_n^{\tau_v}$ will be specific to the sequence of antigenic changes.

If $\mathcal{R}_0^{(n+1)} \leq 1$, then $U_{n+1} = U_n$, otherwise from equation (S9), U_{n+1} is the solution of

$$0 = \frac{1}{R}\ln\left(\frac{U_{n+1}}{U_n}\right) + U_n - U_{n+1} + \sum_{\tau_v}\left(V_n^{\tau_v} - V_{n+1}^{\tau_v}\right) \tag{S12}$$

satisfying $0 < U_{n+1} < U_n$. Then using Equation (S5) we have

$$V_n^{\tau_v} = V_0^{\tau_v}\prod_{i=0}^{n-1}\left(\frac{U_{i+1}}{U_i}\right)^{1-\chi_{|v-i_\tau|}} \tag{S13}$$

where i_τ is the variant at target τ of the strain causing epidemic wave i, and

$$S_n = U_n + \sum_{\tau_v} V_n^{\tau_v} \tag{S14}$$

is the remaining uninfected individuals after the nth epidemic wave. We are therefore equipped to compute the remaining uninfected individuals after the nth epidemic wave for a given sequence of antigenic changes.

Although our derivations have made no assumptions about the 'broadness' of cross-immunity, in what follows we will focus upon the situation in which cross-immunity is limited. We discuss the implications of the 'broadness' of cross-immunity in more depth in Section S10.2.

S4 The likelihood of vaccine escape

Because the outcome, S_n, will depend upon the sequence of antigenic changes, in the main text we focus upon comparing the expected remaining uninfecteds, $\mathbb{E}[S_{\text{final}}]$ for two different scenarios. Specifically, the scenarios we consider are (i) when vaccine escape is rare, and (ii) when vaccine escape is common.

When vaccine escape is rare, we assume that following each epidemic wave, with probability ω a single antigenic change occurs at either target with equal probability, while with probability $1-\omega$, no antigenic change happens. We assume that ω is sufficiently small, that is, vaccine escape is sufficiently rare, such that we can neglect the probability of more than one antigenic change over the time scale of interest. Therefore if we let $S_2(A_jB_k)$ denote the density of hosts without infection-acquired immunity following a second wave by strain A_jB_k, then $\mathbb{E}[S_{\text{final}}]$ is given by

$$\mathbb{E}[S_{\text{final}}] = (1-\omega)S_1 + \omega\left(\frac{1}{2}S_2(A_1B_0) + \frac{1}{2}S_2(A_0B_1)\right) + \mathcal{O}(\omega^2). \tag{S15}$$

When vaccine escape is common, following the initial potential wave by strain A_0B_0, the pathogen will undergo successive one unit antigenic changes (and successive potential waves) until the density of remaining individuals without infection-acquired immunity is less than or equal to $1/R$; at this point, the population will have herd immunity and so any novel strains will be unable to cause an epidemic wave. In this case, $\mathbb{E}[S_{\text{final}}]$ is the average remaining uninfected individuals computed over all possible antigenic sequences terminating in $S_n \le 1/R$. In general this threshold will be reached within 1 or 2 antigenic changes (under the assumption of limited cross-protection, $\chi_{|z|} \approx 0$ for $z > 0$). For epidemics in which vaccine escape is common, we restrict our attention to situations in which we either distribute vaccines between targets, but not variants (so vary x while fixing $y_\tau = 1$), or between variants, but not targets (so vary y_A with $x = 1$).

If we are distributing between targets, a single antigenic change is required to escape vaccination at a given target; after this initial change, any subsequent antigenic change at that target will have no effect. Therefore it suffices to consider two possible epidemic sequences, (1) $A_0B_0 \to A_1B_0 \to A_1B_1$, and (2) $A_0B_0 \to A_0B_1 \to A_1B_1$. We note that other sequences, such as $A_0B_0 \to A_1B_0 \to A_0B_1$, or $A_0B_0 \to A_1B_0 \to A_3B_2$, will be equivalent to one of these two cases. Hence

$$\mathbb{E}[S_{\text{final}}] = \frac{1}{2}S_3(1) + \frac{1}{2}S_3(2), \tag{S16}$$

where $S_3(s)$ is the remaining individuals without infection acquired immunity following the third epidemic wave for epidemic sequence $s = 1$ or $s = 2$. We also note that the two factors of 1/2 in Equations (S15) and (S16) could easily be replaced by weighted probabilities if one epidemic sequence was *a priori* more probable.

If we are instead distributing between variants, since all vaccine doses are directed at target A any antigenic change at target B will have no effect; moreover, it is possible (depending upon y_A) that two antigenic changes at target A will be required to sufficiently deplete the remaining uninfecteds. In combination, this means that $S_3(A_2B_0) = S_4(A_2B_1) = \cdots = S_{3+j}(A_2B_j)$. Thus there is only one antigenic sequence that matters: $A_0B_0 \to A_1B_0 \to A_2B_0$, and so $\mathbb{E}[S_{\text{final}}] = S_3(A_2B_0)$.

An important point to note is that when vaccine escape is common, the best outcome we can hope to achieve is $\mathbb{E}[S_{\text{final}}] = 1/R$. Thus irrespective of vaccine coverage, the population will ultimately be protected by herd immunity acquired through infection. The purpose of vaccination is then to 'ease' the population gradually down to the point of herd immunity so that when herd immunity is reached, there are few active infections in the population. In contrast, rapid epidemic dynamics can lead to 'overshoot' [2], where many active infections exist when herd immunity is reached, meaning that although, on average, infections are not

able to replace themselves through transmission, overall the susceptible fraction is reduced to below $1/R$. Consequently, the vaccine strategy that performs best (i.e., maximises $\mathbb{E}[S_{\text{final}}]$) is that which avoids this overshoot. In contrast, when vaccine escape is rare, considerably better outcomes than $1/R$ can be achieved; for example, it is possible under certain conditions for $\mathbb{E}[S_{\text{final}}] = S_0$.

S5 Epidemic cases

There are two thresholds which delineate the qualitatively different epidemic regimes discussed in the main text. The first is when the epidemic wave by the initial strain, A_0B_0, reduces the remaining uninfecteds to exactly $1/R$, that is, $S_1 = 1/R$. To obtain the threshold value of χ_0 for which this occurs (which we denote $T_{I/II}(p)$), we simultaneously solve equation (S12) when $n = 0$ and equation (S14) when $n = 1$ and $S_1 = 1/R$, that is, we solve

$$\begin{aligned} 0 &= \frac{1}{R}\ln\left(\frac{U_1}{U_0}\right) + U_0 - U_1 + S_0 p\left(1 - \left(\frac{U_1}{U_0}\right)^{1-\chi_0}\right) \\ \frac{1}{R} &= U_1 + S_0 p\left(\frac{U_1}{U_0}\right)^{1-\chi_0} \end{aligned} \tag{S17}$$

for U_1 and χ_0, then set $\chi_0 = T_{I/II}(p)$, which yields

$$T_{\text{I/II}}(p) = 1 - \frac{1}{RS_0 - 1}\ln\left(\frac{RS_0 p}{1 - RS_0(1-p)\mathrm{e}^{-RS_0+1}}\right). \tag{S18}$$

The second threshold occurs when the epidemic wave by the initial strain A_0B_0 is blocked, $\mathcal{R}_0^{(1)} = 1$. To obtain the threshold value of χ_0 for which this occurs (which we denote $T_{II/III}(p)$), we solve

$$\mathcal{R}_0^{(1)} = R(S_0(1-p) + (1-\chi_0)S_0 p) = 1 \tag{S19}$$

for χ_0 and set $\chi_0 = T_{II/III}(p)$ which yields

$$T_{II/III}(p) = \frac{RS_0 - 1}{RS_0 p}. \tag{S20}$$

S6 Metrics to evaluate effectiveness of vaccination

Because vaccination decreases the likelihood of infection, we are primarily interested in reducing the total number of infections over the course of the epidemic. Thus the ‘optimal’ distribution of vaccines across targets and variants is that which maximises $\mathbb{E}[S_{\text{final}}]$. Therefore in the main text we compare the remaining uninfecteds from either distributing across variants, $\mathbb{E}[S_{\text{final}}(x)]$, or distributing across targets, $\mathbb{E}[S_{\text{final}}(y)]$, to conventional vaccination, $\mathbb{E}[S_{\text{final}}(c)]$, that is, we focus upon $\mathbb{E}[S_{\text{final}}(x)] - \mathbb{E}[S_{\text{final}}(c)]$ and $\mathbb{E}[S_{\text{final}}(y)] - \mathbb{E}[S_{\text{final}}(c)]$; when these quantities are positive, then distributing across targets or variants outperforms conventional vaccination.

Another metric we may be interested in is vaccine efficacy, denoted VE. Specifically, we define

$$\text{VE} = 1 - \frac{\text{prob. of infection if vaccinated}}{\text{prob. of infection if unvaccinated}}, \tag{S21}$$

where the probability of infection is calculated over the entire epidemic. Clearly, a higher vaccine efficacy is desirable. If vaccine escape is common, the vaccine efficacy of conventional vaccination is

$$\text{VE}(c) = 1 - \frac{(V_0^{A_0} - V_n^{A_0})/p}{(U_0 - U_n)/U_0}. \tag{S22}$$

If instead vaccine escape is rare, then there are two possible epidemic sequences of interest: (1) $A_0B_0 \to A_1B_0$ and (2) $A_0B_0 \to A_0B_1$. Let $V_n^{\tau_v}(s)$ denote the density of individuals vaccinated against τ_v following the nth epidemic of sequence $s = 1, 2$. Then the expected vaccine efficacy of conventional vaccination is

$$\text{VE}(c) = 1 - \frac{\sum_{s=1,2}\frac{1-\omega}{2}\left(V_0^{A_0}(s) - V_1^{A_0}(s)\right) + \frac{\omega}{2}\left(V_0^{A_0}(s) - V_2^{A_0}(s)\right)}{\sum_{s=1,2}\frac{1-\omega}{2}(U_0(s) - U_1(s)) + \frac{\omega}{2}(U_0(s) - U_2(s))}\frac{U_0}{p}. \tag{S23}$$

A third metric is what we refer to as vaccine matching, denoted VM. Specifically, given a vaccinated individual is infected, what is the probability they were infected by the target/variant that they were vaccinated against (the 'matched' strain)? We calculate this as

$$\mathrm{VM} = \frac{\#\text{ of vaccinated infected by matched strain}}{\#\text{ of vaccinated infected}}, \tag{S24}$$

In general, having a higher VM is desirable because although we have assumed the primary effect of vaccination is to reduce the probability of infection, vaccines often have other effects, such as reducing severity of disease. Our model makes no assumptions about what happens to individuals following infection clearance. Thus although it is possible that individuals fully recover from infection, it is also possible that they suffer long-term health consequences or die due to disease complications. Individuals infected by the strain they were vaccinated against are less likely to suffer from long-term health complications or death. Therefore a higher VM indicates a lower individual risk and is beneficial.

For conventional vaccination, if vaccine escape is common, then the only epidemic sequence that matters is $A_0B_0 \to \cdots \to A_1B_k$ where A_1B_k is the first strain with variant 1 at target A. Thus we can treat this epidemic sequence as $A_0B_0 \to A_1B_0 \to \cdots$, and so

$$\mathrm{VM}(c) = \frac{V_0^{A_0} - V_1^{A_0}}{V_0^{A_0} - V_n^{A_0}}. \tag{S25}$$

If vaccine escape is rare, then as before the two epidemic sequences of interest are (1) $A_0B_0 \to A_1B_0$ and (2) $A_0B_0 \to A_0B_1$. Therefore

$$\mathrm{VM}(c) = \frac{\sum_{s=1,2} \frac{1}{2}(V_0^{A_0}(s) - V_1^{A_0}(s)) + \frac{\omega}{2}(V_1^{A_0}(2) - V_2^{A_0}(2))}{\sum_{s=1,2}\left(\frac{1-\omega}{2}(V_0^{A_0}(s) - V_1^{A_0}(s)) + \frac{\omega}{2}(V_0^{A_0}(s) - V_2^{A_0}(s))\right)}. \tag{S26}$$

S7 Vaccines distributed across targets

When we distribute vaccines across targets, there are two sets of sequences of antigenic changes of interest, $A_0B_0 \to A_1B_0 \to \cdots$ and $A_0B_0 \to A_0B_1 \to \cdots$ (recall that cross-protection is assumed to be limited). The optimal vaccination strategy, x^*, is to distribute vaccine doses so as to maximise protection against one of these sequences (Fig. S1). Without loss of generality, focus upon the set of sequences in which the first antigenic change occurs at target A, that is, $A_0B_0 \to A_1B_0 \to \cdots$.

When vaccine escape is rare, this means choosing $x = x^*$ to block the second epidemic wave by A_1B_0, that is, x^* satisfies $\mathcal{R}_0^{(2)} = 1$. Therefore we solve equation (S12) with $n = 0$ for U_1, that is, we solve

$$0 = \frac{1}{R}\ln\left(\frac{U_1}{U_0}\right) + U_0 - U_1 + S_0p\left(1 - \left(\frac{U_1}{U_0}\right)^{1-\chi_0}\right) \tag{S27}$$

for U_1 and then use that value in

$$1 = R\left(U_1 + S_0p\left(\frac{U_1}{U_0}\right)^{1-\chi_0}(x^* + (1-x^*)(1-\chi_0))\right) = \mathcal{R}_0^{(2)}, \tag{S28}$$

to solve for x^*. Solving the second expression for x^* as a function of U_1 gives

$$x^* = \frac{1}{\chi_0}\left(\frac{1-RU_1}{RS_0p}\left(\frac{U_0}{U_1}\right)^{1-\chi_0} - 1 + \chi_0\right), \tag{S29}$$

while the first equation can be numerically solved for U_1 (there is no analytic solution of U_1 in this case). Since our choice of x does not affect U_1 or S_1, it follows from equation (S15) that x^* will be independent of ω. Of course if x^* is a solution then so too is $1 - x^*$; Fig. S1).

If instead vaccine escape is common (and so epidemics are limited by infection-acquired immunity), then

we want to choose x^* to satisfy $S_2(A_1B_0) = 1/R$. Therefore we solve

$$\begin{aligned}
0 &= \frac{1}{R}\ln\left(\frac{U_1}{U_0}\right) + U_0 - U_1 + S_0 p\left(1 - \left(\frac{U_1}{U_0}\right)^{1-\chi_0}\right) \\
0 &= \frac{1}{R}\ln\left(\frac{U_2}{U_1}\right) + U_1 - U_2 + S_0 p\left(\frac{U_1}{U_0}\right)^{1-\chi_0}\left(1 - x^*\frac{U_2}{U_1} - (1-x^*)\left(\frac{U_2}{U_1}\right)^{1-\chi_0}\right) \\
\frac{1}{R} &= U_2 + S_0 p\left(\frac{U_1}{U_0}\right)^{1-\chi_0}\left(x^*\frac{U_2}{U_1} + (1-x^*)\left(\frac{U_2}{U_1}\right)^{1-\chi_0}\right) = S_2(A_1B_0)
\end{aligned} \tag{S30}$$

for x^*, U_1, and U_2. Note that if we are in case III, that is, $\chi_0 > T_{II/III}(p)$, then $U_1 = U_0 = S_0(1-p)$, and we only need to solve for x^* and U_2. As before, if x^* is optimal, then so is $1 - x^*$ (Fig. S1).

It is straightforward to calculate vaccine efficacy, VE(x), when distributing across targets. When vaccine escape is common, there are two sequences of interest: (1) $A_0B_0 \to A_1B_0 \to \cdots \to A_kB_1$ and (2) $A_0B_0 \to A_0B_1 \to \cdots \to A_1B_k$, where A_1B_k is the first strain with variant A_1 and A_kB_1 is the first strain with variant B_1. Thus we can write these sequences as (1) $A_0B_0 \to A_1B_0 \to A_1B_1$ and (2) $A_0B_0 \to A_0B_1 \to A_1B_1$. Then

$$\mathrm{VE}(x) = 1 - \frac{\sum_{s=1,2}\sum_{\tau_v}(V_0^{\tau_v}(s) - V_n^{\tau_v}(s))/p}{(U_0 - U_n)/U_0}, \quad V_0^{A_0} = px^*,\ V_0^{B_0} = p(1-x^*), \tag{S31}$$

whereas if vaccine escape is rare, there are two possible epidemic sequences, (1) $A_0B_0 \to A_1B_0$ and (2) $A_0B_0 \to A_0B_1$, and so

$$\mathrm{VE}(x) = 1 - \frac{\sum_{s=1,2}\sum_{\tau_v}\frac{1-\omega}{2}\left(V_0^{\tau_v}(s) - V_1^{\tau_v}(s)\right) + \frac{\omega}{2}\left(V_0^{\tau_v}(s) - V_2^{\tau_v}(s)\right)}{\sum_{s=1,2}\frac{1-\omega}{2}(U_0(s) - U_1(s)) + \frac{\omega}{2}(U_0(s) - U_2(s))}\frac{U_0}{p}. \tag{S32}$$

Similarly, we can calculate vaccine matching, VM(x), when distributing across targets. When vaccine escape is common, then

$$\mathrm{VM}(x) = \frac{\sum_{s=1,2}(V_0^{A_0}(s) - V_1^{A_0}(s) + V_0^{B_0}(s) - V_2^{B_0}(s))}{\sum_{s=1,2}(V_0^{A_0}(s) - V_n^{A_0}(s) + V_0^{B_0}(s) - V_n^{B_0}(s))}. \tag{S33}$$

whereas when vaccine escape is rare,

$$\mathrm{VM}(x) = \frac{\sum_{\tau_v}\sum_{s=1,2}\frac{1}{2}(V_0^{\tau_v}(s) - V_1^{\tau_v}(s)) + \frac{\omega}{2}(V_1^{B_0}(1) - V_2^{B_0}(1) + V_1^{A_0}(2) - V_2^{A_0}(2))}{\sum_{\tau_v}\sum_{s=1,2}\left(\frac{1-\omega}{2}(V_0^{\tau_v}(s) - V_1^{\tau_v}(s)) + \frac{\omega}{2}(V_0^{\tau_v}(s) - V_2^{\tau_v}(s))\right)}. \tag{S34}$$

S8 Vaccines distributed across variants

When we distribute vaccines across variants, the two scenarios of vaccine escape are slightly different. First, for epidemics in which vaccine escape is rare, there are two candidate solutions, $y_A = y_{A_0}$, in which we maximise protection against the primary variant at target A, and $y_A = y_{A_1}$, in which we maximise protection against the secondary variant at target A. Specifically,

$$y_{A_0} = \min\left(1, \frac{RS_0 - 1}{RS_0 p\chi_0}\right), \tag{S35}$$

that is, in case II, $y_{A_0} = 1$, while in case III, since $U_1 = U_0$, y_{A_0} is the solution of

$$\mathcal{R}_0^{(1)} = R\left(S_0(1-p) + (1-\chi_0)S_0 p y_{A_0} + S_0 p(1 - y_{A_0})\right) = 1. \tag{S36}$$

In this strategy, any excess doses not needed to block the A_0B_0 epidemic wave are diverted to protect against the (rare) possibility that a strain carrying the variant A_1 emerges (Fig. S2).

When we maximise protection against the secondary variant, $y_A = y_{A_1}$, this means choosing y_{A_1} to satisfy $\mathcal{R}_0^{(2)} = 1$, given the next strain is A_1B_0. That is, we solve

$$\begin{aligned}
0 &= \frac{1}{R}\ln\left(\frac{U_1}{U_0}\right) + U_0 - U_1 + S_0 p y_{A_1}\left(1 - \left(\frac{U_1}{U_0}\right)^{1-\chi_0}\right) + S_0 p(1 - y_{A_1})\left(1 - \frac{U_1}{U_0}\right) \\
1 &= R\left(U_1 + S_0 p y_{A_1}\left(\frac{U_1}{U_0}\right)^{1-\chi_0} + (1-\chi_0)S_0 p(1 - y_{A_1})\frac{U_1}{U_0}\right) = \mathcal{R}_0^{(2)}
\end{aligned} \tag{S37}$$

for y_{A_1} and U_1. Which of y_{A_1} or y_{A_0} performs the best will depend upon R and the likelihood of vaccine escape, ω; when ω is small, then y_{A_0} is best.

Second, for epidemics in which vaccine escape is common, we can always choose y_A to ensure $\mathbb{E}[S_{\text{total}}] = 1/R$. Thus in contrast to distributing vaccine doses between targets, when distributing across variants we can, in theory, always attain the optimum, $\mathbb{E}[S_{\text{final}}] = 1/R$. First, we can choose $y_A = y^*$ such that after the first epidemic $S_1 = 1/R$. To do so, we solve

$$\begin{aligned} 0 &= \frac{1}{R}\ln\left(\frac{U_1}{U_0}\right) + U_0 - U_1 + S_0 p y^* \left(1 - \left(\frac{U_1}{U_0}\right)^{1-\chi_0}\right) + S_0 p(1-y^*)\left(1 - \frac{U_1}{U_0}\right) \\ \frac{1}{R} &= U_1 + S_0 p y^* \left(\frac{U_1}{U_0}\right)^{1-\chi_0} + S_0 p(1-y^*)\frac{U_1}{U_0} \end{aligned} \tag{S38}$$

for U_1 and y^*; doing so gives

$$y^* = \frac{1 - RS_0 \mathrm{e}^{-RS_0+1}}{RS_0 p \mathrm{e}^{-RS_0+1}(\mathrm{e}^{\chi_0(RS_0-1)} - 1)}. \tag{S39}$$

Second, we can choose $y_A = y^\bullet$ such that $S_2 = 1/R$, if we assume the first antigenic change is A_1B_0 (note that this is justified since $U_1 = U_2 = \cdots = U_j$ for A_0B_j, and so we can ignore all antigenic changes to target B). Therefore, we solve

$$\begin{aligned} 0 &= \frac{1}{R}\ln\left(\frac{U_1}{U_0}\right) + U_0 - U_1 + S_0 p y^\bullet \left(1 - \left(\frac{U_1}{U_0}\right)^{1-\chi_0}\right) + S_0 p(1-y^\bullet)\left(1 - \frac{U_1}{U_0}\right) \\ 0 &= \frac{1}{R}\ln\left(\frac{U_2}{U_1}\right) + U_1 - U_2 + S_0 p y^\bullet \left(\frac{U_1}{U_0}\right)^{1-\chi_0}\left(1 - \frac{U_2}{U_1}\right) + S_0 p(1-y^\bullet)\frac{U_1}{U_0}\left(1 - \left(\frac{U_2}{U_1}\right)^{1-\chi_0}\right) \\ \frac{1}{R} &= U_2 + S_0 p y^\bullet \left(\frac{U_1}{U_0}\right)^{1-\chi_0}\frac{U_2}{U_1} + S_0 p(1-y^\bullet)\frac{U_1}{U_0}\left(\frac{U_2}{U_1}\right)^{1-\chi_0} \end{aligned}$$

for U_1, U_2 and $y^\bullet$. Although both y^* and $y^\bullet$ maximise $\mathbb{E}[S_{\text{final}}]$, from a public health perspective $y^\bullet$ is the superior option as it maximises $\mathbb{E}[S_{\text{final}}]$ after at least one antigenic change, and so two waves, rather than one epidemic and no antigenic change.

It is straightforward to calculate vaccine efficacy, $\mathrm{VE}(y)$, when distributing across variants. When vaccine escape is common, there is one sequence of interest, $A_0B_0 \to \cdots \to A_1B_k \to \cdots \to A_2B_k$, which we can write as $A_0B_0 \to A_1B_0 \to A_2B_0$. Thus

$$\mathrm{VE}(y) = 1 - \frac{(V_0^{A_0} - V_n^{A_0} + V_0^{A_1} - V_n^{A_1})/p}{(U_0 - U_n)/U_0}, \quad V_0^{A_0} = py^\bullet,\ V_0^{A_1} = p(1-y^\bullet), \tag{S40}$$

whereas if vaccine escape is rare, there are two possible epidemic sequences, (1) $A_0B_0 \to A_1B_0$ and (2) $A_0B_0 \to A_0B_1$, and so

$$\mathrm{VE}(y) = 1 - \frac{\sum_{s=1,2}\sum_{\tau_v} \frac{1-\omega}{2}\left(V_0^{\tau_v}(s) - V_1^{\tau_v}(s)\right) + \frac{\omega}{2}\left(V_0^{\tau_v}(s) - V_2^{\tau_v}(s)\right)}{\sum_{s=1,2} \frac{1-\omega}{2}(U_0(s) - U_1(s)) + \frac{\omega}{2}(U_0(s) - U_2(s))}\frac{U_0}{p}. \tag{S41}$$

Similarly, we can calculate vaccine matching, $\mathrm{VM}(y)$, when distributing across variants. When vaccine escape is common, then

$$\mathrm{VM}(y) = \frac{V_0^{A_0} - V_1^{A_0} + V_1^{A_1} - V_2^{A_2}}{V_0^{A_0} - V_n^{A_0} + V_0^{A_1} - V_n^{A_1}}. \tag{S42}$$

whereas when vaccine escape is rare

$$\mathrm{VM}(y) = \frac{\sum_{s=1,2} \frac{1}{2}(V_0^{A_0}(s) - V_1^{A_0}(s)) + \frac{\omega}{2}(V_1^{A_0}(2) - V_2^{A_0}(2) + V_1^{A_1}(1) - V_2^{A_1}(1))}{\sum_{\tau_v}\sum_{s=1,2}\left(\frac{1-\omega}{2}(V_0^{\tau_v}(s) - V_1^{\tau_v}(s)) + \frac{\omega}{2}(V_0^{\tau_v}(s) - V_2^{\tau_v}(s))\right)}. \tag{S43}$$

S9 Vaccines distributed across targets and variants simultaneously

Finally, consider distributing vaccines across both targets and variants. When we are free to choose all three of (x, y_A, y_B), many different combinations can lead to similar S_n. For example, when antigenic change is not limiting, we have already shown that when only manipulating y_A (with $x = 1$) there are two values of y_A which ensure the optimal outcome, $\mathbb{E}[S_{\text{final}}] = 1/R$. Therefore we have the opportunity to apply further constraints to identify optimal combinations. One approach would be to apply additional metrics. For example, in addition to maximising S_n we could also seek to maximise the average n at which S_n is first equal to $1/R$ (this was what separated $y^\bullet$ from y^*), or minimise the variance between epidemic sequences (when $x = 1/2$ and $y_A = y_B$). Alternatively, we may impose the constraint that we are free to distribute across targets (x) and then across both variants in the same way; that is, we take $y_A = y_B = y$ and so we are choosing the pair (x, y) rather than the triplet (x, y_A, y_B). The motivation for imposing this constraint is that doing so allows us to see when either distribution dimension has primacy. Specifically, when distributing between targets is more important, then the optimal (x, y) will show variation between targets as coverage (p and χ_0) changes, while all the doses will be allocated towards the primary variant, $y = 1$. In particular, we expect to see optimal values x^* and $1 - x^*$ where $x^* \neq 1/2$, as observed when distributing across targets only. When distributing between variants is more important, then we should expect y to vary with vaccine coverage while doses will be evenly allocated across targets, $x = 1/2$.

The results are much as we would expect (Fig. S3). When the probability of antigenic change is low (ω small), it is best to distribute between targets while focusing upon the primary antigen, that is, choose $y = 1$ and $x = x^*$ where x^* is provided in equation (S29). As the likelihood of antigenic change increases, however, it becomes optimal to distribute doses between variants, that is, $y < 1$, with doses equally allocated between targets, $x = 1/2$. Specifically, if allocating between variants is the preferred strategy (and so $x = 1/2$), then the optimal choice of y will block the next epidemic wave by either strain A_1B_0 or A_0B_1, that is, y satisfies $\mathcal{R}_0^{(2)} = 1$ (since $x = 1/2$, $\mathcal{R}_0^{(2)}$ is not sensitive to which strain emerges). Therefore we solve

$$0 = \frac{1}{R}\ln\left(\frac{U_1}{U_0}\right) + U_0 - U_1 + S_0py\left(1 - \left(\frac{U_1}{U_0}\right)^{1-\chi_0}\right) + S_0p(1-y)\left(1 - \frac{U_1}{U_0}\right) \tag{S44}$$

$$1 = R\left(U_1 + (1-\chi_0)\frac{S_0py}{2}\left(\frac{U_1}{U_0}\right)^{1-\chi_0} + \frac{S_0py}{2}\left(\frac{U_1}{U_0}\right)^{1-\chi_0} + (1-\chi_0)\frac{S_0p(1-y)}{2}\frac{U_1}{U_0} + \frac{S_0p(1-y)}{2}\frac{U_1}{U_0}\right) = \mathcal{R}_0^{(2)} \tag{S45}$$

for U_1 and y (note the 1/2 multiplier is because it is equally likely that strain A_1B_0 or A_0B_1 will emerge). This can be done with a computer algebra package, but as the answer can only be expressed in terms of the Lambert W function, we do not show it here. Note that as we approach the threshold between case I and II, that is, $T_{I/II}(p)$, the solution of (S44) in terms of y will exceed 1; in this case the optimal solution is $y = 1$ and we resort to varying x (i.e., choosing x^* from equation (S29); Fig. S3).

S10 Model extensions and limitations

Our model made a number of simplifying assumptions. Here we identify the limitations of our approach, and ask how relaxing the assumptions associated with these limitations affects the value of mosaic vaccination.

In what follows, whenever we make specific calculations, we take the optimal distributions obtained from the simple model and ask if relaxing any of the assumptions reduces the success of mosaic vaccination below that of conventional vaccination.

S10.1 Mutations

Antigenic change was assumed to arise in our model without consideration of its source. This is reasonable if antigenic novelty originates outside the focal population. For example, it may come from a reservoir animal population or be otherwise imported from a source population (e.g., influenza A H3N2 from southeast Asia [3, 4]). If instead we focused strictly on antigenic change from *de novo* mutation in the focal population, then there are multiple additional considerations.

First, it may be better to 'hit hard' using conventional vaccination in the hopes of preventing antigenic change, rather than to distribute vaccine doses in anticipation of an unlikely mutation. This is particularly true in case III. So in some circumstances, if antigenic change is generated by *de novo* mutation, our model may underestimate the efficacy of conventional vaccination.

Second, we assumed that each wave consists of a single strain. Although this may be reasonable if antigenic variation originates elsewhere, it is less likely when variation is generated by mutations in the focal population. If our results were extended to include multi-strain waves, the predicted efficacy of conventional vaccination would be weakened, since whenever an escape mutant arises during an ongoing wave, there are more individuals without infection-acquired immunity and so available to be infected by the escape mutant. This would lead to a larger wave by the strain with limited vaccine protection.

Finally, the other complicating factor when explicitly considering mutation is whether or not vaccination can induce within-host evolution, biasing which target is most likely to generate novel variants. Although consideration of this possibility is beyond the scope of the current work, in at least some relevant diseases (e.g., influenza A), modelling suggests within-host evolution due to vaccination plays a limited role [5–7].

S10.2 Broadness of cross-protection

We assumed cross-protection is limited, such that $\chi_z = 0$ for $z > 0$. Under this 'all-or-nothing' model of protection, we can use the breadth of protection to define what constitutes a new variant, as described in the main text. To compare vaccines with broader (but not universal) protection, the key change in parameters would then be that vaccine escape is less likely. Thus, our results for the situation in which vaccine escape is rare would apply to more broadly protective vaccines.

But how would these results change if we relaxed the 'all-or-nothing' nature of vaccine protection, and instead allowed for a more complex cross-protection function? The shape, or 'broadness' (i.e., how slow the decay of χ_z is for increasing z), of the cross-protection function is of no consequence in case I. In cases II and III, however, the primary role of the broadness of vaccine cross-immunity is that it determines the reduction in vaccine protection against new strains. If vaccine cross-immunity is broad, antigenic change will cause a small reduction in vaccine protection, and so a smaller subsequent wave. If, however, cross-immunity is narrow, antigenic change will cause a large reduction in vaccine protection, and so potentially a large wave. Thus increasing cross-protection increases the efficacy of conventional vaccination in case II and III. For example, if antigenic change is not limiting, conventional vaccination is more likely to achieve the optimum of $\mathbb{E}[S_{\text{final}}] = 1/R$ with broad cross-immunity, whereas with narrow cross-immunity, substantial overshoot is more likely. In our model, the shape of cross-immunity will ultimately depend upon the units of antigenic space. For example, if k_i and k_{i+1} differ by a single, nearly-neutral mutation, cross-protection will tend to be broad. If instead antigenic variation arises in a source population with pre-existing immunity, large differences between k_i and k_{i+1} may be necessary to escape extinction in the source population, and so cross-protection may be narrow.

S10.3 Waning natural immunity

We assumed that naturally-acquired immunity was broad and long-lasting, at least over the timescale of interest. However, it is possible that naturally-acquired immunity may wane between successive epidemic waves. For example, it is believed influenza A induces a broadly cross-protective immune response lasting roughly a year, decaying to a more narrowly protective response in the long-term [8]. Equally, it is possible that natural immunity primarily confers strong protection against the infection strain, and limited protection against other strains; and so individuals infected by the previous epidemic waves may return to the susceptible class between waves. Therefore how does incorporating waning immunity affect our predictions?

Suppose there are only two possible epidemic waves, and thus two possible sequences of antigenic change, $A_0B_0 \to A_1B_0$ and $A_0B_0 \to A_0B_1$. After the first epidemic wave, a fraction γ individuals infected during the first wave return to the susceptible, unvaccinated class, that is, if U_1^* is the fraction of susceptibles following the first wave, and after waning immunity has taken place, then

$$U_1^* = U_1 + \gamma\left(U_0 - U_1 + \sum_{\tau_v}(V_0^{\tau_v} - V_1^{\tau_v})\right). \tag{S46}$$

In this scenario, using the remaining uninfected individuals as an evaluative metric is less informative, since individuals may be infected twice. Therefore consider the total number of infections across both waves.

In general, mosaic vaccination is more effective at reducing the number of infections (Fig. S4). This is logical: mosaic vaccination either reduces vaccine protection against the second epidemic wave of one epidemic sequence (distributing between targets) or the initial variant (distributing between variants) so as to increase protection against the second epidemic wave of the other sequence or against a future variant, respectively. Waning immunity will act to increase the size of the second epidemic wave. Thus creating more balanced protection across the sequences (distributing between targets) or stronger protection against the second wave (distributing between variants) is advantageous.

S10.4 Evolution of other life-history traits

The successive epidemic waves in our model are caused by antigenically varying strains. However, pathogen evolution need not be constrained to antigenic evolution; life-history evolution, due to selection or chance, is also possible. For example, following the first epidemic wave, in addition to antigenic changes, subsequent strains may be more (or less) transmissible, all else being equal, than the initial strain. If life-history evolution does occur, but we cannot predict its likelihood or direction, how robust is mosaic vaccination?

Suppose vaccine escape is common, and that following the first epidemic wave by the initial strain (with transmissibility β), the transmissibility of all subsequent, antigenically distinct strains is $\beta(1+\sigma)$. Thus the initial strain may be more, $-1 \leq \sigma < 0$, or less, $\sigma > 0$, transmissible than all subsequent strains. If the subsequent strains are more transmissible than the initial, mosaic vaccination outperforms conventional vaccination. If the subsequent strains are less transmissible than the initial strain, mosaic vaccination is no worse than conventional vaccination when distributing between targets, but when distributing between variants (using strategy $y^{\bullet}$ rather than y^{*}), mosaic vaccination can be either better (smaller $|\sigma|$) or worse (larger $|\sigma|$) than conventional vaccination (Fig. S5).

The logic is straightforward. Mosaic vaccination allocates doses so as to provide more robust protection against future epidemic waves. If these waves are by more transmissible strains, then the value of mosaic vaccination increases. If the future waves are by less transmissible strains, allocating more doses against future variants can be wasteful, particularly if the future variants are much less transmissible. These results equally apply to evolutionary changes in duration of carriage, δ, since the quantity of importance is $R = \beta\delta$.

S10.5 Breadth vs. strength of immunity

As described in Section S10.2, each individual vaccine dose was restricted to a single target and variant combination. It is assumed that this is the limit of what can be safely included in a single vaccine, where the limit is due to, for example, cross-reactivity, immune interference or other safety considerations.

However, what if mosaic vaccination is compared to a 'universal' vaccine which provides broad, but weakened, protection against all variants? For example, some of the candidates for universal influenza vaccines may provide less robust protection against each strain then a 'normal' vaccine does against its target strain [9–11]. How large must the reduction in peak protection of universal vaccines be before mosaic vaccination is a superior option? To answer this, suppose S_{mos} is the best outcome achievable by mosaic vaccination for a vaccine of efficacy χ_0 and coverage p, and suppose λ controls the reduced efficacy of universal vaccines, that is, the efficacy of a universal vaccine is $\chi_{|i-j|} = \lambda\chi_0$ for all i, j. Let λ^* denote the value of λ at which the remaining uninfecteds for universal vaccination is the same as S_{mos}. Then λ^* is the solution of

$$0 = \frac{1}{R}\ln\left(\frac{U_1}{1-p}\right) + 1 - p - U_1 + p\left(1 - \left(\frac{U_1}{1-p}\right)^{1-\lambda^*\chi_0}\right), \tag{S47}$$

$$S_{\text{mos}} = U_1 + p\left(\frac{U_1}{1-p}\right)^{1-\lambda^*\chi_0}. \tag{S48}$$

Following some algebra,

$$\lambda^* = \frac{1}{\chi_0}\left(1 - \frac{1}{R(1-S_{\text{mos}})}\ln\left(\frac{p}{S_{\text{mos}} - (1-p)\mathrm{e}^{-R(1-S_{\text{mos}})}}\right)\right). \tag{S49}$$

In general, λ^* is a decreasing function of p and χ_0 (Fig. S6). Thus as vaccine coverage and protection increases, universal vaccines have to provide increasingly weak protection to be outperformed by mosaic vaccination, as we should expect.

S10.6 Reduced vaccine efficacy against variants or targets

Our base model assumes that all vaccines are equally effective against their target strain. In practice, this need not be true. For example, variants may have immune evasive properties reducing vaccine efficacy, or vaccine efficacy may be compromised because of an inability to predict future vaccine strains. Likewise, we assumed that both targets were of equal efficacy. Yet, in many cases identifying equally efficacious targets may not be possible; for example, antibodies against the hemagglutinin and neuramindiase proteins in influenza may not provide comparable protection [12, 13]. Therefore, how will reduced vaccine efficacy against variants, or reduced vaccine efficacy against target B, affect mosaic vaccination?

To answer this, we focus upon the case in which vaccine escape is common, and consider two situations:

(1) **Vaccines against future variants are less effective.** Here, vaccines directed against all variants $i > 0$ have protection $(1-c)\chi_0$, $0 \leq c \leq 1$, against their target variant, and we are distributing across variants, using the optimal distribution when $c = 0$.

(2) **Vaccines against target B are less effective.** Here, vaccines directed towards any variant at target B (so B_i for all $i \geq 0$) have reduced protection $(1-c)\chi_0$, $0 \leq c \leq 1$, and we are distributing across targets using the optimal distribution when $c = 0$ (using the distribution that allocates more doses to target A).

Numerical simulations indicate that in either of these situations, even when $c = 1$, mosaic vaccination still does no worse than conventional vaccination for either scenario (Fig. S7).

The logic is as follows. When we are in case II or III (and so mosaic and 'conventional' vaccination differ) and vaccine escape is common, then following the first epidemic wave, substantial overshoot of herd immunity can occur for conventional vaccination [14]. Thus even if doses distributed to the other target or to future variants are wasted ($c = 1$), there can still be a benefit, i.e., reducing overshoot through a larger initial wave and subsequently smaller susceptible pool for the second wave. Of course, as $c \to 0$, these doses will be wasted less and less, and so mosaic vaccination becomes better and better. Notice that when vaccines are less effective against future variants, increasing c will decrease the advantage of mosaic vaccination, but when vaccines are less effective against target B, changing c can increase or decrease the advantage of mosaic vaccination, depending upon p and χ_0 (Fig. S7). This is because when distributing across variants, the optimal distribution ensures that $\mathbb{E}[S_{\text{final}}] = 1/R$ (when $c = 0$); increasing c will worsen this performance. However, when distributing across targets, the optimal distribution need not attain $\mathbb{E}[S_{\text{final}}] = 1/R$, and so increasing c can, in certain circumstances, improve the performance of mosaic vaccination (Fig. S7).

References

[1] MATLAB, 2019 *version 9.6.0 (R2019a)*. Natick, Massachusetts: The MathWorks Inc.

[2] Handel, A., Longini, I. & Antia, R., 2007 What is the best control strategy for multiple infectious disease outbreaks? *Proc. R. Soc. B.* **274**, 833–837.

[3] Bedford, T., Riley, S., Barr, I., Broor, S., Chadha, M., Cox, N., Daniels, R., Gunasekaran, C., Hurt, A., Kelso, A. *et al.*, 2015 Global circulation patterns of seasonal influenza viruses vary with antigenic drift. *Nature* **532**, 217–220.

[4] Russell, C. A., Jones, T. C., Barr, I. G., Cox, N. J., Garten, R., Gregory, V., Gust, I. D., Hampson, A. W., Hay, A. J., Hurt, A. C. *et al.*, 2008 The global circulation of seasonal influenza A (H3N2) viruses. *Science* **320**, 340–346.

[5] Morris, D. H., Petrova, V. N., Rossine, F. W., Parker, E., Grenfell, B. T., Neher, R., Levin, S. A. & Russell, C. A., 2020 Asynchrony between virus diversity and antibody selection limits influenza virus evolution. *bioRxiv* (doi:https://doi.org/10.1101/2020.04.27.064915).

[6] Dinis, J. M., Florek, N. W., Fatola, O. O., Moncla, L. H., Mutschler, J. P., Charlier, O. K., Meece, J. K., Belongia, E. A. & Friedrich, T. C., 2016 Deep sequencing reveals potential antigenic variants at low frequencies in influenza A virus-infected humans. *J. Virol* **90**, 3355–65.

[7] Debbink, K., McCrone, J. T., Petrie, J. G., Truscon, R., Johnson, E., Mantlo, E. K., Monton, A. S. & Lauring, A. S., 2017 Vaccination has minimal impact on the intrahost diversity of H3N2 influenza viruses. *PLoS Pathogens* **13**, e1006194.

[8] Kucharski, A., Lessler, J., Cummings, D. A. T. & Riley, S., 2018 Timescales of influenza A/H3N2 antibody dynamics. *PLoS Biol.* **16**, e2004974.

[9] Viboud, C., Gostic, K., Nelson, M. I., Price, G. E., Perofsky, A., Sun, K., Sequeira Trovão, N., Cowling, B. J., Epstein, S. L. & Spiro, D. J., 2020 Beyond clinical trials: Evolutionary and epidemiological considerations for development of a universal influenza vaccine. *PLOS Pathogens* **16**, e1008583. (doi: 10.1371/journal.ppat.1008583).

[10] Arinaminpathy, N., Ratmann, O., Koelle, K., Epstein, S. L., Price, G. E., Viboud, C., Miller, M. A. & Grenfell, B. T., 2012 Impact of cross-protective vaccines on epidemiological and evolutionary dynamics of influenza. *Proc. Natl. Acad. Sci.* **109**, 3173–3177.

[11] Subramanian, R., Graham, A. L., Grenfell, B. T. & Arinaminpathy, N., 2016 Universal or specific? A modeling-based comparison of broad-spectrum influenza vaccines against conventional, strain-matched vaccines. *PLOS Computational Biology* **12**, e1005204. (doi:10.1371/journal.pcbi.1005204).

[12] Wong, S., Waite, B., Ralston, J., Wood, T., Reynolds, G. E., Seeds, R., Newbern, E. C., Thompson, M. G., Huang, Q. S. & Webby, R. J., 2020 Hemagglutinin and neuraminidase antibodies are induced in an age- and subtype-dependent manner after influenza virus infection. *J. Virol.* **94**, e01385–19.

[13] Weiss, C. D., Wang, W., Lu, Y., Billings, M., Eick-Cost, A., Couzens, L., Sanchez, J. L., Hawksworth, A. W., Seguin, P., Myers, C. A. *et al.*, 2019 Neutralizing and neuraminidase antibodies correlate with protection against influenza during a late season A/H3N2 outbreak among unvaccinated military recruits. *Clinical Infectious Diseases* **71**, 3096–3102. (doi:10.1093/cid/ciz1198).

[14] Zarnitsyna, V. I., Bulusheva, I., Handel, A., Longini, I. M., Halloran, M. E. & Antia, R., 2018 Intermediate levels of vaccination coverage may minimize seasonal influenza outbreaks. *PLOS ONE* **13**, e0199674. (doi:10.1371/journal.pone.0199674).

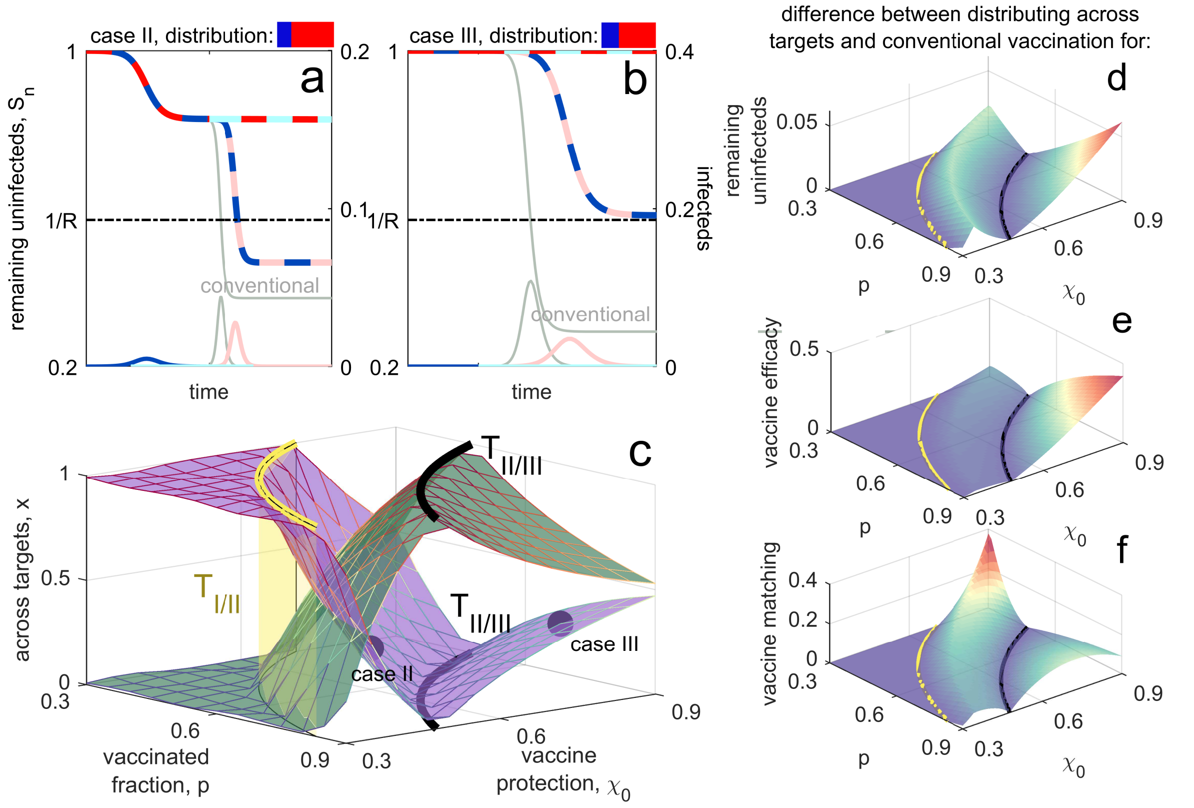

Figure S1: Optimal distribution of vaccines across targets when vaccine escape is rare. Panels **a** and **b** show example epidemic dynamics for cases II and III, respectively, with conventional vaccination (light grey) shown for reference. Note that following the initial epidemic wave by strain A_0B_0, there are two possible sequences of interest, $A_0B_0 \to A_1B_0$ and $A_0B_0 \to A_0B_1$; both are shown. The optimal distribution across targets (shown in panel **c** as vaccine coverage, p, and protection, χ_0, vary) maximises the remaining susceptibles averaged over these sequences; when x is optimal, so is $1-x$ (purple, green). Panels **d-f** show how distributing across targets outperforms conventional vaccination for three metrics; positive values indicate the degree to which mosaic vaccination is superior. Panel **d**, shows the difference in remaining uninfecteds, $\mathbb{E}[S_{\text{final}}]$. Panel **e** shows the difference in vaccine efficacy, measured as attack rate over the course of the epidemic. Panel **f** shows the difference in vaccine matching, defined as the probability that an individual that is both vaccinated and infected is infected by a strain that they were vaccinated against. In all panels, $R = 1.75$ and the thresholds $T_{I/II}(p)$ (yellow surface) and $T_{II/III}(p)$ (black lines) are included for reference; beyond the yellow surface, vaccine efficacy is sufficiently low that evolution has no effect on the epidemic, and any distribution of vaccines between targets will produce the same outcome (for a given p and χ_0).

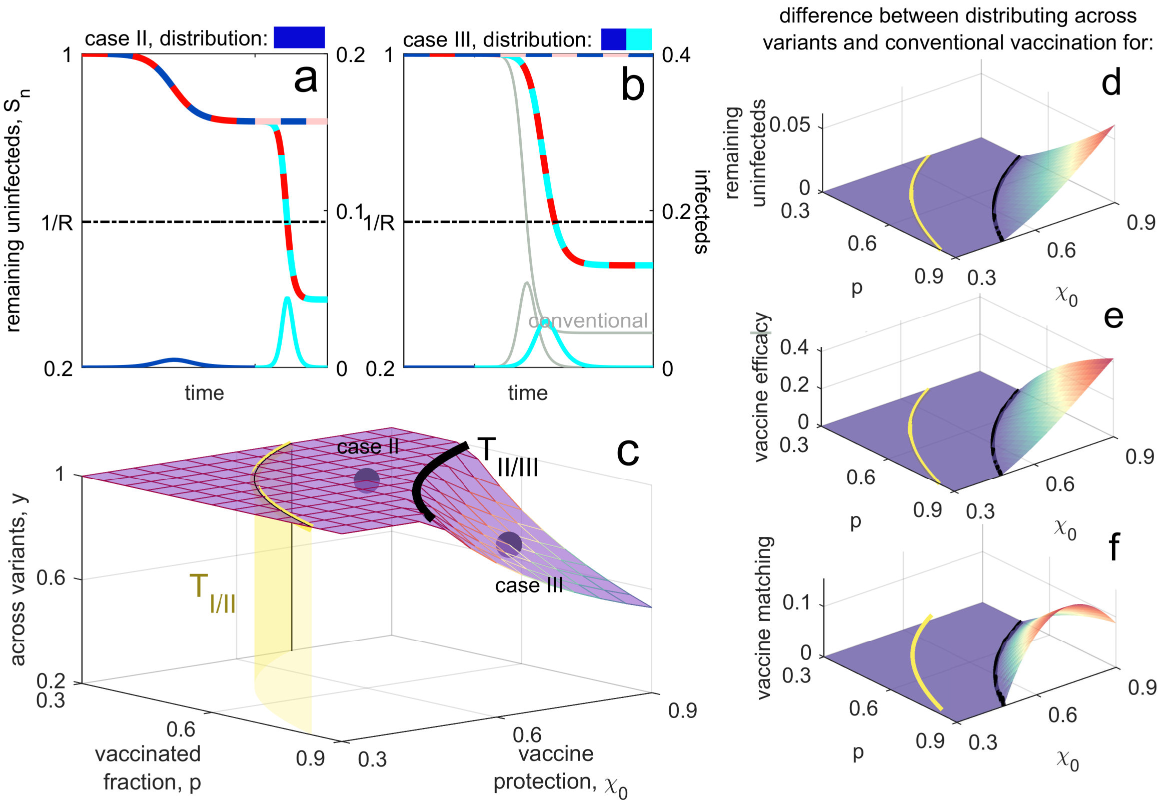

Figure S2: Optimal distribution of vaccines across variants when vaccine escape is rare. Panels **a** and **b** show example epidemic dynamics for cases II and III, respectively, with conventional vaccination (light grey) shown for reference (in panel **a**, the optimal distribution *is* conventional vaccination). Following the initial epidemic wave by strain A_0B_0, there are two possible sequences of interest, $A_0B_0 \to A_1B_0$ and $A_0B_0 \to A_0B_1$; both are shown. The optimal distribution across variants (shown in panel **c** as vaccine coverage, p, and protection, χ_0, vary) maximises the remaining susceptibles averaged over these sequences. Panels **d-f** show how distributing across variants outperforms conventional vaccination for three metrics; positive values indicate the degree to which mosaic vaccination is superior. Panel **d** shows the difference in remaining uninfecteds, $\mathbb{E}[S_{\text{final}}]$. Panel **e** shows the difference in vaccine efficacy, measured as attack rate over the course of the epidemic. Panel **f** shows the difference in vaccine matching, defined as the probability that an individual that is both vaccinated and infected is infected by a strain that they were vaccinated against. In all panels, $R = 1.75$ and the thresholds $T_{I/II}(p)$ (yellow surface) and $T_{II/III}(p)$ (black lines) are included for reference; beyond the yellow surface, vaccine efficacy is sufficiently low that evolution has no effect on the epidemic, and any distribution of vaccines between targets will produce the same outcome (for a given p and χ_0).

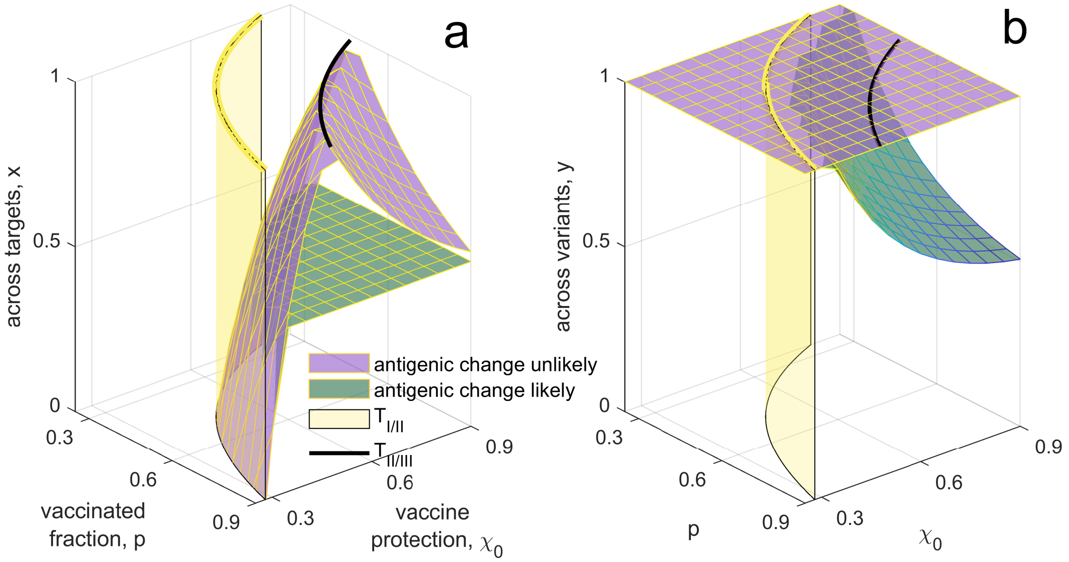

Figure S3: Optimal distribution of vaccines when distributing between targets (panel **a**) and variants (panel **b**) simultaneously. In both panels, the purple surface corresponds to the optimal distribution when antigenic change is unlikely (i.e., ω is small) while the green surface corresponds to the optimal distribution when antigenic change is likely. As before, beyond the yellow surface, vaccine efficacy is sufficiently low that evolution has no effect on the epidemic outcome. In general, when antigenic change is unlikely (purple surfaces) we should distribute between targets and protect against the primary variant ($y = 1$), whereas when antigenic change is likely (green surfaces) we should distribute between variants with doses evenly allocated between targets ($x = 1/2$). Note that for visual clarity in panel **a**, we have only shown one possible optimal solution; but if x is optimal, then so is $1 - x$. In both panels, $R = 1.75$.

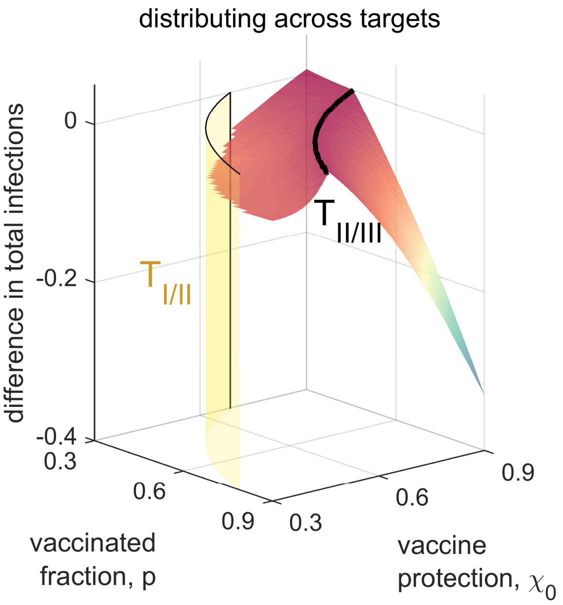

Figure S4: **Effect of waning natural immunity.** Here we show the difference in total infections between mosaic vaccination and conventional vaccination over two epidemic waves when a fraction $\gamma = 0.75$ of individuals infected during the first wave lose immunity and are susceptible at the beginning of the second wave. Mosaic vaccination is either distributing between targets (panel **a**) or variants (panel **b**). The more negative the value, the fewer the infections for mosaic vaccination as compared to conventional vaccination. In both panels, $R = 1.7$. Note that mosaic vaccination uses the optimal strategy obtained when only two epidemic waves are possible (but assuming $\gamma = 0$).

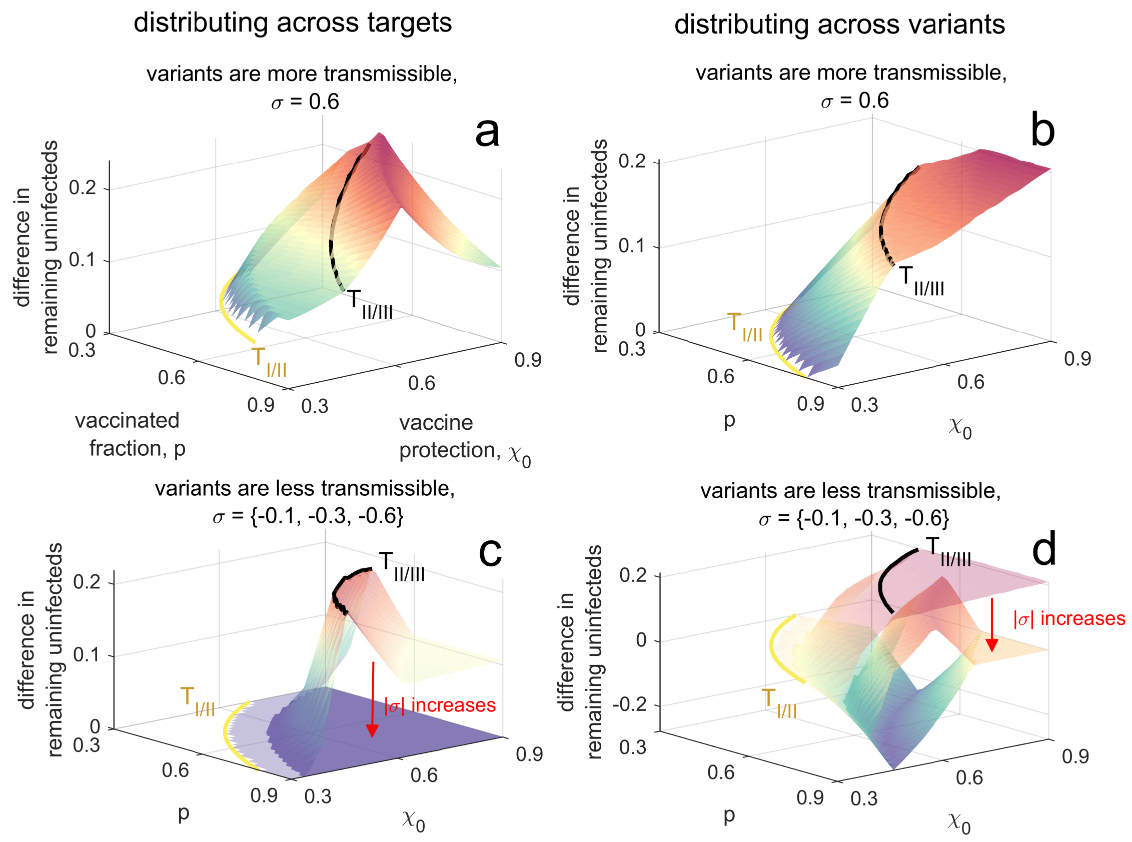

Figure S5: **Outcome when life-history evolution occurs simultaneously with antigenic evolution.** Here we suppose that following the first epidemic wave, by a strain with $R = 1.7$, the subsequent strains have $R = 1.7(1+\sigma)$, for $\sigma = 0.6$ (panels **a**,**b**) or $\sigma = \{-0.1, -0.3, -0.6\}$ (panels **c**,**d**). Each panel shows the difference in remaining uninfecteds between mosaic vaccination and conventional vaccination, where mosaic vaccination is either distributing between targets (panels **a**,**c**) or variants (panels **b**,**d**). Only when the variants are significantly less transmissible and we are distributing between variants, will life-history evolution undo the advantage of mosaic vaccination. In all cases, the mosaic vaccination distribution (either across targets or variants) is the optimal distribution when $\sigma = 0$ and vaccine escape is common.

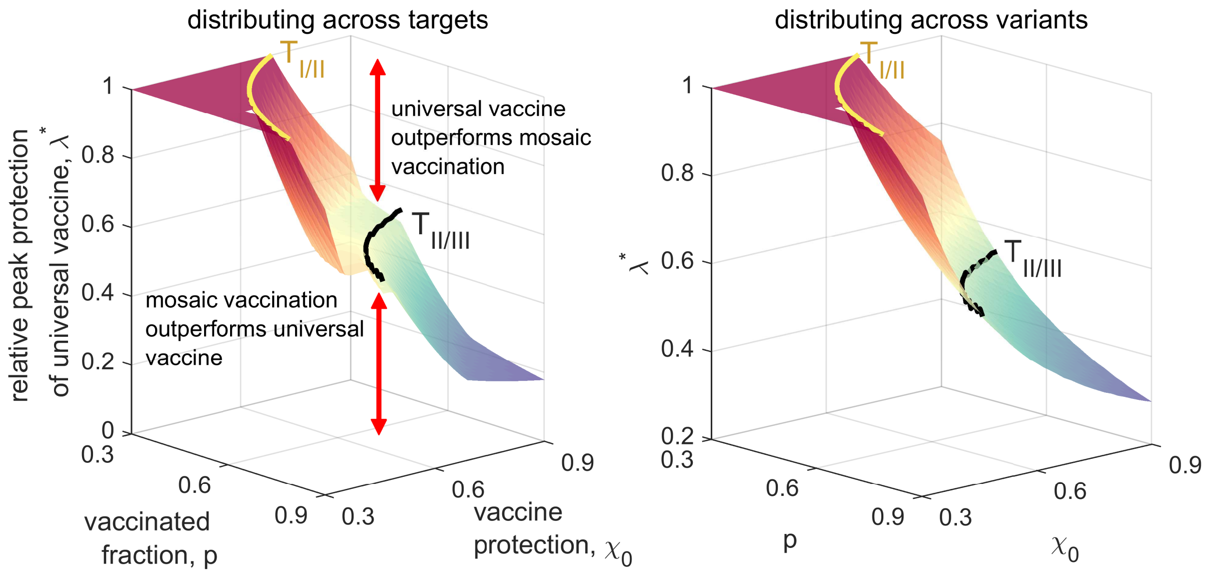

Figure S6: **Peak protection vs broad cross-protection.** Here we show how high the peak protection conferred by a universal vaccine must be for it to outperform mosaic vaccination. For all values of λ lower than the plotted surface, mosaic vaccination is superior to universal vaccination. In both panels, $R = 1.7$, and vaccine escape is common.

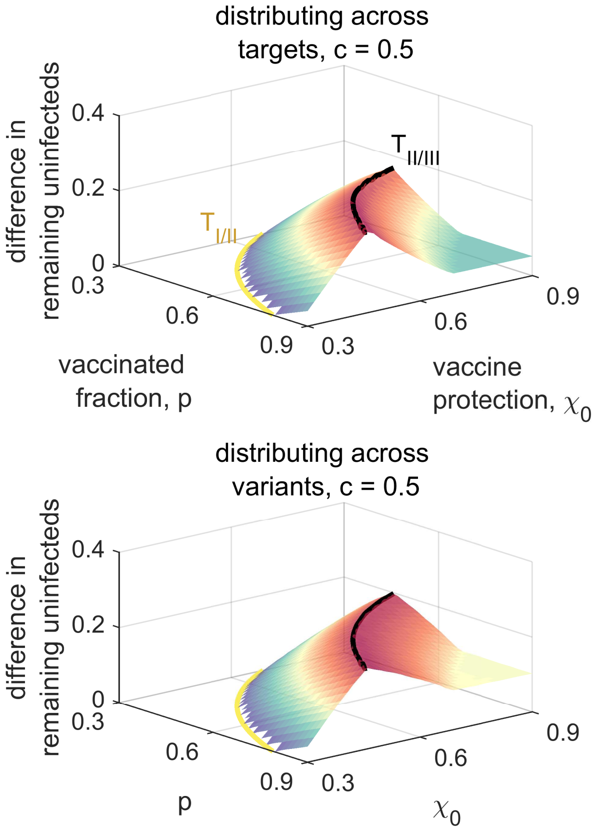

Figure S7: **Mosaic vaccination can still outperform conventional vaccination when targets provide unequal protection or protection against variants is reduced.** If vaccines have reduced protection, $(1-c)\chi_0$, against target B or future variants, mosaic vaccination is still more effective than conventional vaccination. In all panels, $R = 1.7$, vaccine escape is common, and the mosaic vaccination distribution is the optimal distribution when $c = 0$.
